# Hyperinflammatory environment drives dysfunctional myeloid cell effector response to bacterial challenge in COVID-19

**DOI:** 10.1371/journal.ppat.1010176

**Published:** 2022-01-10

**Authors:** Srikanth Mairpady Shambat, Alejandro Gómez-Mejia, Tiziano A. Schweizer, Markus Huemer, Chun-Chi Chang, Claudio Acevedo, Judith Bergada-Pijuan, Clément Vulin, Daniel A. Hofmaenner, Thomas C. Scheier, Sanne Hertegonne, Elena Parietti, Nataliya Miroshnikova, Pedro D. Wendel Garcia, Matthias P. Hilty, Philipp Karl Buehler, Reto A. Schuepbach, Silvio D. Brugger, Annelies S. Zinkernagel

**Affiliations:** 1 Department of Infectious Diseases and Hospital Epidemiology, University Hospital of Zurich, University of Zurich, Zurich, Switzerland; 2 Institute of Intensive Care, University Hospital of Zurich, University of Zurich, Zurich, Switzerland; Columbia University, UNITED STATES

## Abstract

COVID-19 displays diverse disease severities and symptoms including acute systemic inflammation and hypercytokinemia, with subsequent dysregulation of immune cells. Bacterial superinfections in COVID-19 can further complicate the disease course and are associated with increased mortality. However, there is limited understanding of how SARS-CoV-2 pathogenesis and hypercytokinemia impede the innate immune function against bacterial superinfections. We assessed the influence of COVID-19 plasma hypercytokinemia on the functional responses of myeloid immune cells upon bacterial challenges from acute-phase COVID-19 patients and their corresponding recovery-phase. We show that a severe hypercytokinemia status in COVID-19 patients correlates with the development of bacterial superinfections. Neutrophils and monocytes derived from COVID-19 patients in their acute-phase showed an impaired intracellular microbicidal capacity upon bacterial challenges. The impaired microbicidal capacity was reflected by abrogated MPO and reduced NETs production in neutrophils along with reduced ROS production in both neutrophils and monocytes. Moreover, we observed a distinct pattern of cell surface receptor expression on both neutrophils and monocytes, in line with suppressed autocrine and paracrine cytokine signaling. This phenotype was characterized by a high expression of CD66b, CXCR4 and low expression of CXCR1, CXCR2 and CD15 in neutrophils and low expression of HLA-DR, CD86 and high expression of CD163 and CD11b in monocytes. Furthermore, the impaired antibacterial effector function was mediated by synergistic effect of the cytokines TNF-α, IFN-γ and IL-4. COVID-19 patients receiving dexamethasone showed a significant reduction of overall inflammatory markers in the plasma as well as exhibited an enhanced immune response towards bacterial challenge *ex vivo*. Finally, broad anti-inflammatory treatment was associated with a reduction in CRP, IL-6 levels as well as length of ICU stay and ventilation-days in critically ill COVID-19 patients. Our data provides insights into the transient functional dysregulation of myeloid immune cells against subsequent bacterial infections in COVID-19 patients and describe a beneficial role for the use of dexamethasone in these patients.

## Author summary

COVID-19 caused by SARS-CoV-2 induces immune-paralysis characterized by misdirected host responses and altered levels of inflammatory mediators. Additionally, bacterial superinfections can further exacerbate the disease. Here, we report an in-depth functional characterization of the effector response and phenotypic properties of neutrophils and monocytes derived from critically ill COVID-19 patients towards bacterial superinfection. We show that elevated levels of specific cytokine clusters, positively correlate with the development of bacterial superinfections in these patients. Neutrophils and monocytes of critically ill COVID-19 patients showed impaired bactericidal capacity, which was mediated by elevated inflammatory mediators in the plasma. The observed impaired bactericidal capacity in critically ill COVID-19 patients was due to reduced classical effector functions of neutrophils and monocytes. Interestingly, lower levels of overall inflammatory mediators as well as reduction in length of ICU stay and ventilation-days in critically ill COVID-19 patients treated with dexamethasone was observed. These data suggest that the exhaustion and paralysis of the cellular innate immunity against bacterial challenge in critically ill COVID-19 patients is driven by a misdirected host-response, characterized by a hyperinflammatory environment including dysregulated cytokine levels. These results further emphasize the utility and importance of dampening these inflammatory mediators via broad anti-inflammatory therapy in COVID-19 patients, which improves the antibacterial effector functions of neutrophils and monocytes.

## Introduction

The Coronavirus-disease-2019 (COVID-19), caused by the severe acute respiratory syndrome coronavirus 2 (SARS-CoV-2) can affect multiple organs and important functional units, such as the respiratory or the circulatory system. The clinical presentation of COVID-19 is diverse and varies widely. While most patients exhibit only mild to moderate symptoms, approximately 10% to 15% of patients progress to a severe disease state [1]. This severe course of COVID-19 may require intensive care unit (ICU) support, including mechanical ventilation [2], and is characterized by acute respiratory distress syndrome (ARDS) as well as cardiovascular, gastrointestinal and neurological dysfunctions [3–7].

Furthermore, COVID-19 patients have been reported to show a complex immune dysregulation, characterized by misdirected host responses, altered levels of inflammatory mediators [8–11] including impaired interferon-mediated antiviral response [12,13], as well as high plasma cytokine levels [12,14,15]. These high levels of both pro- and anti-inflammatory cytokines including IL-6, IL-10, IFNs and TNF-α are believed to induce functional paralysis of the immune cells [16–18]. This hypercytokinemia is usually associated with mobilization of myeloid immune cells such as neutrophils and monocytes, further augmenting the tissue damage, thereby suggesting that the hypercytokinemia might be associated with disease severity [11,16,19]. In addition, multiple studies have reported neutrophil hyperactivation, dysfunctional mature neutrophils and emergency granulopoiesis resulting in efflux of immature cells from the bone marrow in critically ill COVID-19 patients [20–22]. Apart from the resulting neutrophilia, severe COVID-19 is characterized by lymphopenia and broad myeloid immune cell-dysregulation [6,9,10,22–25]. Several recent studies have proposed emergency myelopoiesis as a response to severe viral infections, including activation of myeloid progenitor cells in the bone marrow, which can lead to release of suppressive immature neutrophils during COVID-19 and is associated with severe disease manifestations [20,22,26,27].

Beyond the systemic dysregulation in severe COVID-19, bacterial superinfections are associated with increased morbidity and mortality [5,8,9,28–30]. In our prospective single centre cohort study, we showed that 42.2% of ICU COVID-19 patients suffered from bacterial superinfections [31]. These were associated with reduced ventilator-free days (VFDs) at 28 days and significantly increased ICU length of stay [31]. However, there is a limited understanding of how SARS-CoV-2 immunopathogenesis impedes innate immune signaling and function against secondary bacterial infections. Similarly, the role of myeloid immune cells and their ability to respond to bacterial infections during severe COVID-19 remains to be elucidated.

Recent clinical trials demonstrated that treatment with the corticosteroid dexamethasone in COVID-19 patients with ARDS resulted in improved clinical outcome [32]. The significant mortality benefit and immunomodulatory effects of corticosteroids in critically ill COVID-19 patients indicate that COVID-19 pathophysiology is associated with a broadly dysregulated inflammatory response, which potentially impairs antibacterial effector functions of myeloid immune cells. Hence, a detailed understanding of this altered immunological state and its effect on the innate immune response against secondary bacterial infections during severe COVID-19 is crucial for the development of effective therapeutic strategies.

Here, we aimed to investigate the functional response and phenotypic properties of neutrophils and monocytes derived from critically ill COVID-19 patients during their acute- and subsequent recovery (rec)-phase towards bacterial challenge as well as the signaling mediators underlying this response.

## Results

### Extensive COVID-19-mediated hypercytokinemia correlates with subsequent bacterial superinfections

We first assessed the plasma levels of cytokines involved in neutrophil and monocyte functional responses in a subset of patients from our prospective cohort of critically ill COVID-19 ICU patients (acute, n = 25), including the same patients in their rec-phase (rec, n = 19) (S1 and S2 Tables) as well as healthy donors (n = 17). As shown previously [12,33,34], we observed that the levels of cytokines affecting neutrophil function, such as G-CSF, IL-8, IL-4, MIP-1α, MIP-2α, MIP-1β and SDF-1α, were significantly increased in acute-phase patients as compared to rec-phase and healthy donors. These levels decreased upon recovery and were similar to values measured in healthy donors (S1 Fig). For monocyte effectors, we found the most significant changes between acute- and rec-phase as well as healthy donors in the levels of CX_3_CL_1_, IP10 and MCP-1 (S1 Fig).

**Fig 1:**
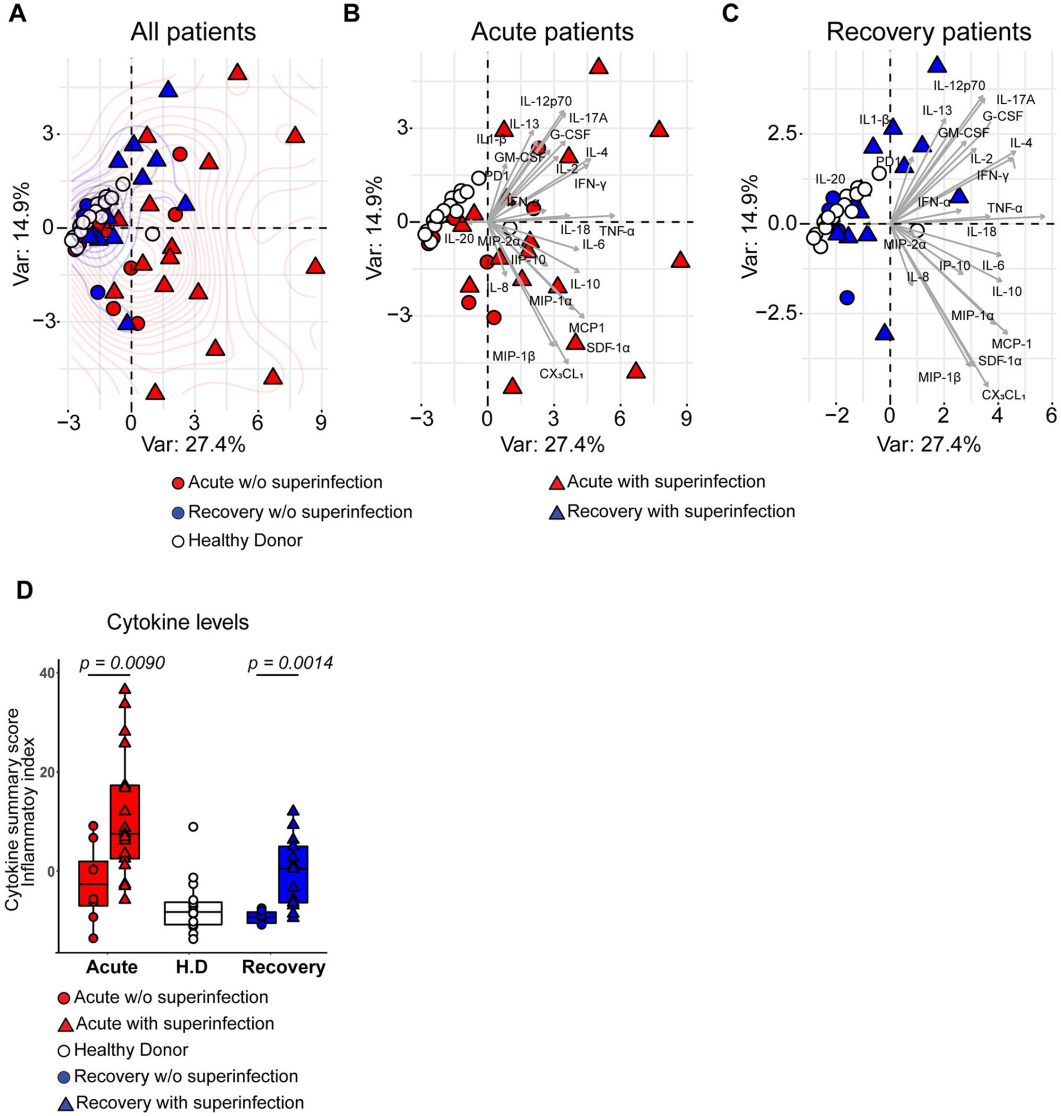
Characterization of inflammatory mediators in COVID-19 plasma and its association with bacterial superinfection. (**A**) PCA of healthy donors (white, n = 17), acute- (red, n = 25) and rec- (blue, n = 19) phase COVID-19 patients grouping the plasma cytokine levels and status of secondary bacterial infections (superinfection). Patients who developed or had bacterial superinfection at the time of sampling are depicted as triangle and patients without (w/o) superinfection as circle symbols. (**B**) PCA of healthy donors (white, n = 17) and acute-phase patients (red, n = 25). Patients who developed bacterial superinfection are depicted as triangle and patients without (w/o) superinfection as circle symbols. (**C**) PCA of healthy donors (white, n = 17), and rec-phase patients (blue, n = 19). Patients with superinfection are depicted as triangle and patients without superinfection as circle symbols. (**D**) Normalized cytokine values (sum of Z-scores) in the plasma of acute (red), rec (blue) patients with or without (w/o) bacterial superinfection and healthy donors (white). Data presented as box plots with box indicating interquartile range and error bars indicating highest and lowest value. For panel D, p values were determined by using Mann-Whitney test.

In a next step, we sought to investigate whether the cytokine levels in COVID-19 patients who developed secondary bacterial infections (bacterial superinfection) differed as compared to patients who did not develop a bacterial superinfection during their stay in the ICU. Principal component analysis (PCA) showed that cytokines measured in the acute and rec-phase of COVID-19 patients clustered apart from healthy donors (Fig 1A). Both, acute- (Fig 1B) and rec-phase COVID-19 patients (Fig 1C) who developed a bacterial superinfection displayed increased separation on the density curve as compared to those without (Fig 1A–1C). This was confirmed by calculating the normalized cytokine values (sum of Z-scores), termed cytokine summary score (inflammatory index), in the plasma. Patients who developed a bacterial superinfection showed significantly elevated cumulative cytokine levels (inflammatory index) in both acute- and rec-phase (Fig 1D). To rule out the effects of ongoing bacterial superinfection during the acute-phase sampling, a further analysis was performed after excluding four acute-phase patients who already presented with bacterial superinfection during the time of acute-phase sampling (ICU admission). This analysis is in line with the finding, that extensive hypercytokinemia and a significantly elevated inflammatory index was mainly observed in COVID-19 patients who were to develop a bacterial superinfection during their stay in the ICU (both acute- and rec-phase) (S2A–S2D Fig). Furthermore, integrative correlation mapping of clinical parameters taken within 24 to 48 hours from blood and plasma sampling revealed that cytokine levels positively correlated with the bacterial superinfection status, which in turn positively correlated with ICU length of stay and ventilation days (S2E Fig) [31]. Overall, extensive COVID-19 hypercytokinemia correlated with the development of subsequent bacterial superinfections.

### Reduced clearance of intracellular bacteria by neutrophils and monocytes of acute-phase COVID-19 patients

**Fig 2:**
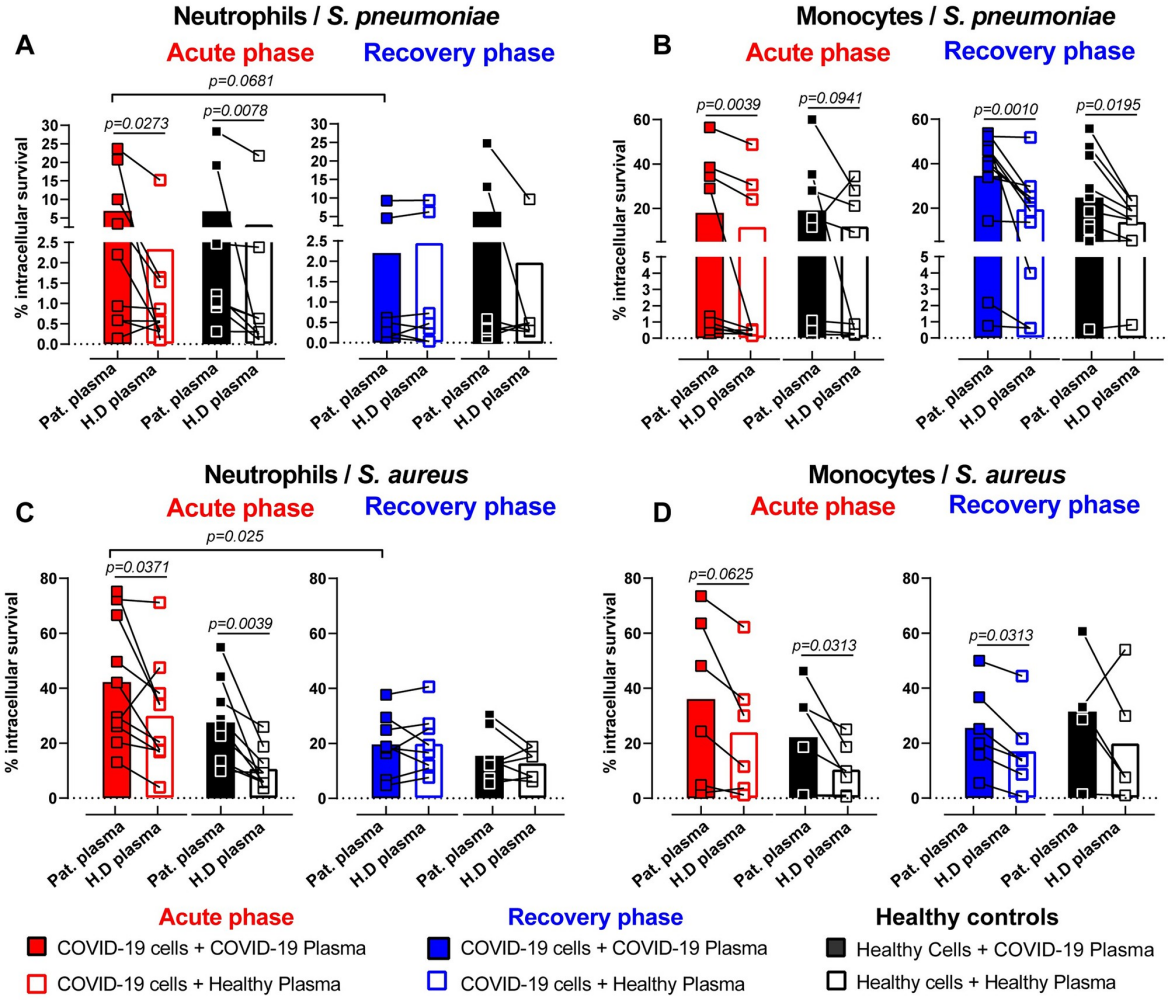
Impaired intracellular bactericidal capacity of innate immune cells in acute-phase COVID-19 patients. **(A-D)** Intracellular killing capacity of COVID-19 patient (acute-phase: red and rec-phase: blue) or healthy donor neutrophils **(A and C)** and monocytes **(B and D)** (n = 8–10) pre-exposed to 10% patient plasma (solid symbols) or healthy donor plasma (open symbols) for 3 h and subsequently infected with SP **(A and B)** or SA **(C and D)** at MOI 10. Neutrophils and monocytes were isolated freshly from blood and stimulated with the respective plasma conditions. Each symbol represents cells from one human subject, stimulated with either COVID-19 or healthy donor plasma from one donor. P values were determined by using Wilcoxon signed-rank test without adjustment for multiple testing.

Aiming to further dissect these findings, we assessed the neutrophil and monocyte function upon bacterial challenge *ex vivo*. Neutrophils and monocytes derived from critically ill COVID-19 patients or healthy donors were incubated with either autologous or heterologous plasma prior to bacterial challenge with *Streptococcus pneumoniae* (SP) or *Staphylococcus aureus* (SA) (Fig 2A–2D and S3A–S3H Fig). Neutrophils from acute-phase patients internalized significantly less SP when stimulated with their own as compared to healthy donor plasma (S3A Fig). We did not observe significant differences in the phagocytosis ability of monocytes challenged with SP (S3B Fig). Similarly, no plasma or cell-mediated effect on phagocytosis ability was observed when either neutrophils or monocytes were challenged with SA (S3C and S3D Fig).

We observed that neutrophils and monocytes from COVID-19 patients or healthy donors incubated with acute-phase COVID-19 plasma had impaired intracellular bactericidal function with a significant reduction in their ability to clear bacteria as compared to healthy plasma stimulation (Fig 2A–2D). Additionally, a direct comparison of acute- to rec-phase COVID-19 neutrophils and monocytes revealed increased intracellular bacterial survival in acute-phase neutrophils but not monocytes (S3E–S3H Fig). Stimulation of COVID-19 or healthy donor neutrophils with COVID-19 rec-phase plasma did not show any impairment in their ability to eliminate intracellular bacteria compared to healthy plasma stimulation (Fig 2A and 2C). In contrast, monocytes from rec-phase patients still displayed reduced bacterial killing capacity (Fig 2B and 2D and S3F Fig). These findings were further corroborated by stimulating healthy donor monocytes with acute-phase COVID-19 plasma, showing significantly impaired ability to clear intracellular bacteria as compared to stimulation with rec-phase or healthy donor plasma (S3I and S3J Fig). These data suggested that soluble plasma factors during acute-phase COVID-19 impairs the intracellular microbicidal ability of neutrophils and monocytes.

### Impaired neutrophil effector responses against bacterial challenges in acute-phase COVID-19 patients

**Fig 3:**
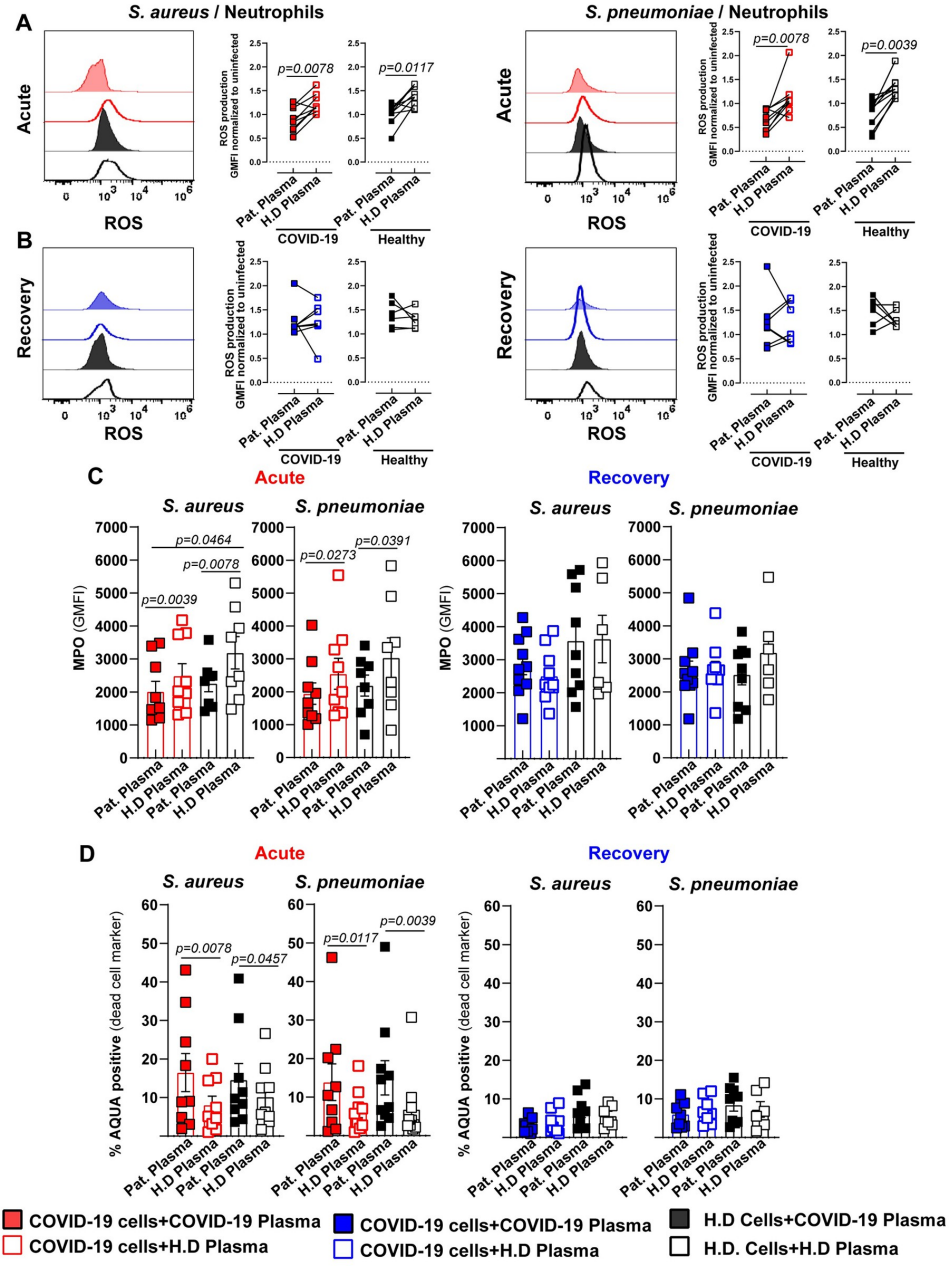
Impaired neutrophil effector response against bacterial challenge in acute COVID-19 patients. Functional characterization of COVID-19 patient’s (acute-phase: red and rec-phase: blue) or healthy donor’s neutrophils pre-exposed to 10% COVID-19 plasma (solid symbols) or healthy plasma (open symbols) for 3 h and subsequently challenged with either SA or SP at MOI 1. (**A and B**). Neutrophil functionality was assessed by quantification of ROS in acute- (**A**) and rec-phase patients (**B**) (n = 7–8), intracellular MPO (**C**) (right: acute-phase) (left: rec-phase) and cell viability (**D**) (right: acute-phase) (left: rec-phase) (n = 7–9) and compared to healthy donors. Each symbol represents cells from one human subject, exposed to either COVID-19 or healthy donor plasma from one donor. In C and D, data are presented as the mean value ± SEM. For panels A and B, p values were determined by using Wilcoxon signed-rank test, for panel C and D by Mann-Whitney test.

To assess the factors involved in the reduced intracellular killing capacity of acute-phase COVID-19 neutrophils, we analyzed key neutrophil effector responses such as reactive oxygen species (ROS) and myeloperoxidase (MPO) production, cell death and neutrophil extracellular traps (NETs) formation. Stimulation of neutrophils from acute-phase patients or healthy donors with acute-phase COVID-19 plasma resulted in significantly lower levels of ROS upon bacterial challenge compared to stimulation with healthy plasma (Fig 3A, SA (left) and SP (right)). Conversely, stimulation of neutrophils from rec-phase patients or healthy donors with rec-phase plasma showed no such effect (Fig 3B, SA (left) and SP (right)).

Additionally, stimulation of neutrophils from acute patients and healthy donors with acute-phase patient plasma resulted in significantly lower levels of MPO compared to stimulation with healthy plasma upon bacterial challenge (Fig 3C (left) and S4A and S4B Fig). In line with the normalized ROS levels during rec-phase, neutrophils from rec-phase stimulated with autologous plasma exhibited MPO levels comparable to cells stimulated with healthy plasma, after bacterial challenge (Fig 3C, right). Since increased rates of dysregulated cell death of various cell types during COVID-19 have been described [35,36], we investigated whether neutrophils from critically ill COVID-19 patients showed an increased tendency towards cell death during bacterial infection. Neutrophils stimulated with acute-phase COVID-19 plasma, irrespective of their origin, showed increased cell death (S4C Fig). Moreover, upon subsequent bacterial challenge cell death was further augmented (Fig 3D (left) and S4D Fig). In contrast, neutrophils from rec-phase patients or healthy donors stimulated with auto- or heterologous plasma prior to bacterial challenge showed no difference in their viability (Fig 3D, right).

**Fig 4:**
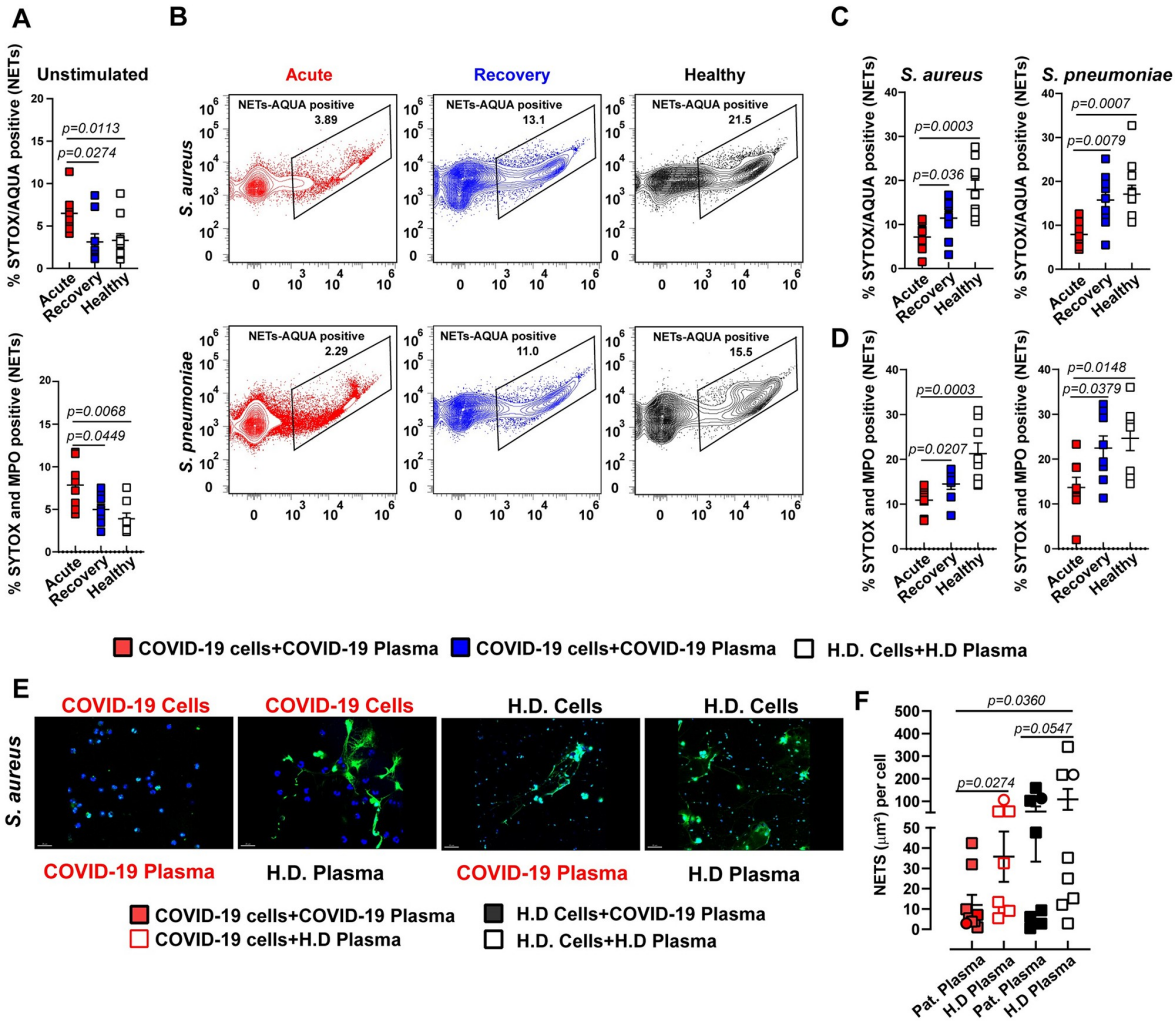
Abrogated classical NETs production in COVID-19 patients upon secondary bacterial challenge. (**A**) Flow cytometry analysis of SYTOX and AQUA positive neutrophils (top) and MPO-SYTOX positive neutrophils (bottom) isolated from fresh blood from acute- or rec-phase COVID-19 patients or healthy donors exposed to 10% autologous plasma for 3 h (n = 6–8). (**B-D**) SYTOX and AQUA positive neutrophils (top) and MPO-SYTOX positive neutrophils (bottom) from acute- or rec-phase COVID-19 patients or healthy donors pre-exposed to 10% autologous plasma for 3 h and subsequently challenged with SA or SP at MOI 1 for 1 h (n = 6–8). (**E**) Representative confocal images of NETs formation by neutrophils from acute-phase COVID-19 patients or healthy donors pre-stimulated with 10% acute-phase COVID-19 or healthy plasma and subsequently challenged with SA at MOI 10 for 1.5 h. Neutrophils were stained by HOECHST (nuclei: blue) and SYTOX (extracellular DNA: green). (**F**) Quantification of NETs using fluorescence microscopy (squares, n = (226–8898)) and confocal microscopy (circles, n = (19–113)) nuclei per point (n = 7–8). Each symbol represents cells from a single COVID-19 patient or healthy donor exposed to either COVID-19 or healthy donor plasma from one donor. In A, C, D, and F, data are presented as the mean value ± SEM. For panels A, C, D and F, p values were determined by using Mann-Whitney test.

Recently, it has been proposed that NETs contribute to the formation of microthrombi in COVID-19 and that serum from COVID-19 patients triggered NETs release by healthy neutrophils [37,38]. Since NETs formation is a strategy to eliminate extracellular pathogens [39], we tested the hypothesis that bacterial challenge-mediated cell death of neutrophils isolated from acute-phase patients is due to increased classical NETs release. Neutrophils from acute-phase patients exhibited a higher amount of spontaneous extracellular DNA-release (Fig 4A (top) and S7A Fig) and elevated levels of MPO-DNA complexes (Fig 4A (bottom) and S7B Fig) than neutrophils from rec-phase patients or healthy donors. However, bacterial challenge resulted in significantly lower release of NETs and MPO-DNA complexes from neutrophils derived from acute-phase patients as compared to neutrophils from rec-phase patients or healthy donors (Fig 4B–4D). The inability to release classical NETs upon bacterial challenge was confirmed by fluorescence microscopy (Fig 4E and 4F and S5A and S5B Fig), thereby suggesting that bacteria-induced neutrophil cell death was not due to increased classical NETs release. Together, these data suggest that neutrophils from acute-phase COVID-19 patients are in a state of exhaustion, resulting in reduced production of ROS, MPO and classical NETs release upon secondary bacterial challenge.

### Functional impairment of classical monocyte subset against bacterial challenge in COVID-19 patients

Next, we examined the functional response of monocytes from COVID-19 patients, which can play a substantial role against bacterial challenge. Analysis of monocyte subset frequencies based on CD14 and CD16 expression revealed significantly lower proportions of classical (CD14+ CD16-) monocytes during acute COVID-19. Similarly, non-classical (CD14dim CD16+) monocyte proportions were reduced during both acute- and rec-phase COVID-19 compared to healthy donors (S6A Fig). Classical monocytes from acute-phase patients as well as healthy donors stimulated with plasma from acute-phase patients resulted in significantly lower levels of ROS in response to bacterial challenge as compared to stimulation with healthy plasma (S6B–S6D Fig). In line, classical monocytes from rec-phase patients stimulated with autologous plasma also produced lower levels of ROS as compared to stimulation with healthy plasma (S6C–S6D Fig). Whereas stimulation of monocytes with acute-phase plasma as well as with healthy plasma had no effect on the production of nitric oxide (NO), another effector molecule, upon challenge with bacteria (S6E–S6F Fig). There was no difference in ROS levels in non-classical monocytes from acute- and rec-phase patients as well as healthy donors after challenge with SA (S6G Fig). On the other hand, upon SP challenge, non-classical monocytes from rec-phase patients produced higher levels of ROS when stimulated with healthy plasma as compared to autologous plasma (S6H Fig). Together, these data suggest that classical monocytes were skewed towards significantly impaired ROS, but not NO, production.

### Neutrophil signaling alterations in acute-phase COVID-19 patients further characterizes a dysfunctional phenotype

**Fig 5:**
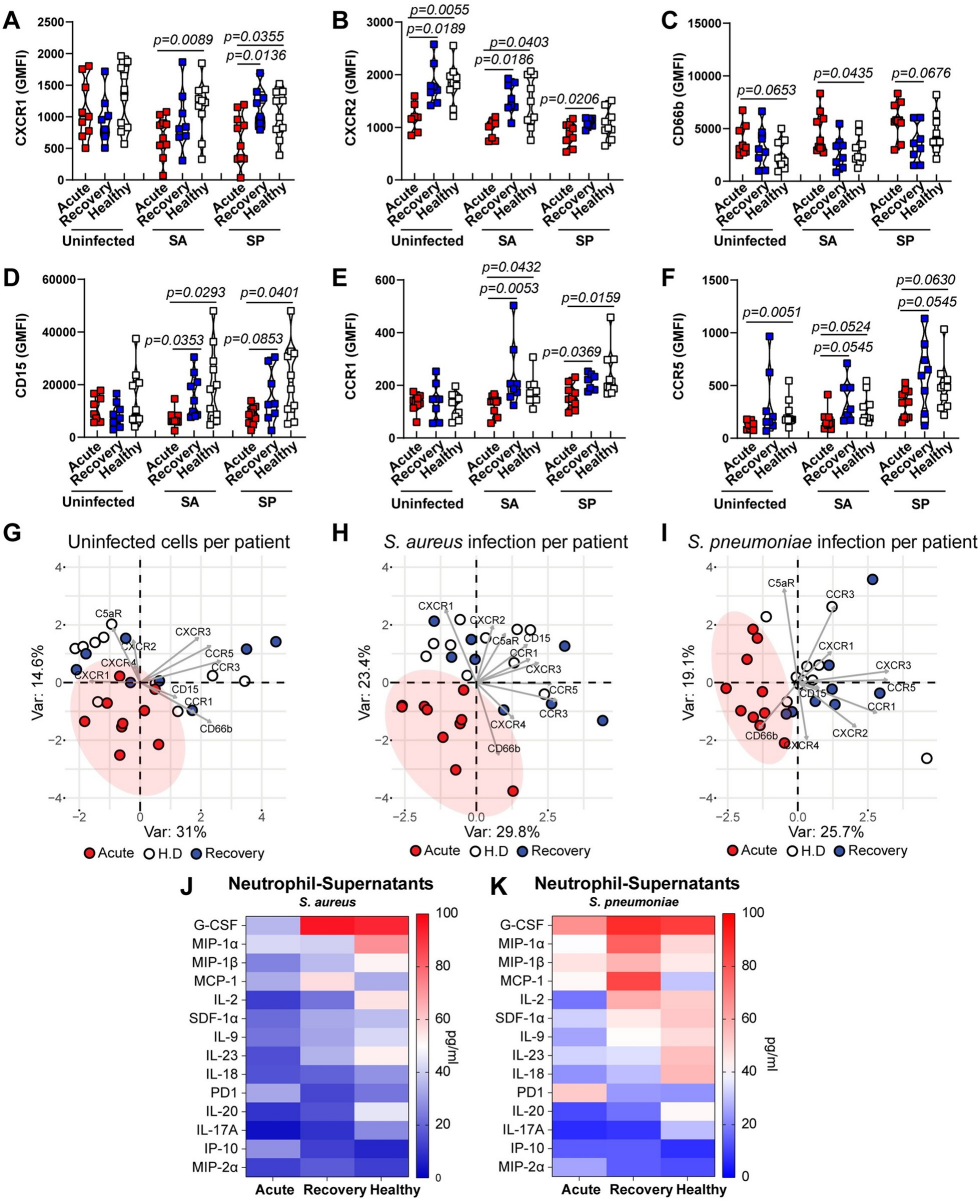
Expression of surface markers and secretion of cytokines in neutrophils upon bacterial challenge. (**A-F**) Expression of key surface markers of neutrophils from acute or rec-phase COVID-19 patients or healthy donors pre-exposed to 10% autologous plasma for 3 h and subsequently either challenged with SA or SP at MOI 1 or left unchallenged (n = 8–10). (**G-I**) PCA of cell surface phenotype of COVID-19 patients acute- (red) or rec-phase (blue) and healthy donors’ (white) neutrophils at the basal level without bacterial challenge (**G**), upon SA infection (**H**) or SP infection (**I**). Each symbol represents cells from a single COVID-19 patient or healthy donor exposed to either COVID-19 or healthy donor plasma. (**J and K**). Heat map of cytokines secreted by neutrophils from COVID-19 patients (acute- and rec-phase) and healthy controls after bacterial challenge with SA (**J**) or SP (**K**) (n = 3–4). For panels A-F, p values were determined by using Mann-Whitney test.

Given the observed impaired neutrophil effector response to bacterial challenges in acute-phase COVID-19 patients, we investigated potentially pivotal signaling mechanisms and receptor phenotypes of neutrophils. Neutrophils from acute-phase patients showed a significant decrease in the expression of the chemokine receptors CXCR2, CXCR3 and CCR5 (Fig 5B and 5F and S7C Fig). Additionally, we observed higher levels of the activation marker CD66b in neutrophils from acute-phase patients (Fig 5C). Furthermore, upon bacterial challenge, a similar pattern of cell surface receptor phenotype with reduced expression of CXCR1, CXCR2, CXCR3, CCR1, CCR5 and the maturation marker CD15, with increased expression of CXCR4 and CD66b was found in neutrophils from acute-phase COVID-19 patients (Fig 5A–5F and S7C–S7F Fig). Overall, PCA of acute-phase COVID-19 patients showed clear separation from rec-phase patients and healthy donors, exhibiting a strong clustering for their receptor phenotype, whereas rec-phase and healthy controls largely overlapped (Fig 5G–5I). Finally, we studied whether this distinct neutrophil phenotype in COVID-19 patients was also characterized by impaired cytokine production involved in the autocrine-paracrine signaling upon bacterial challenge. We found that acute COVID-19 neutrophils secreted reduced G-CSF, MIP-1α, MIP-1β, MCP-1, IL-2, SDF-1α, IL-9, IL-17A, IL-18, IL-20 and IL-23 levels, but secreted increased soluble PD1, IP-10 and MIP-2α levels compared to rec-phase and healthy neutrophils upon bacterial challenge (Fig 5J–5K). Collectively, these data suggest that acute COVID-19 is marked by the presence of immature hyper-activated, dysfunctional neutrophils, displaying reduced effector responses upon secondary bacterial challenge.

### Monocyte subpopulation signaling alterations in COVID-19 characterize impaired antibacterial phenotype

**Fig 6:**
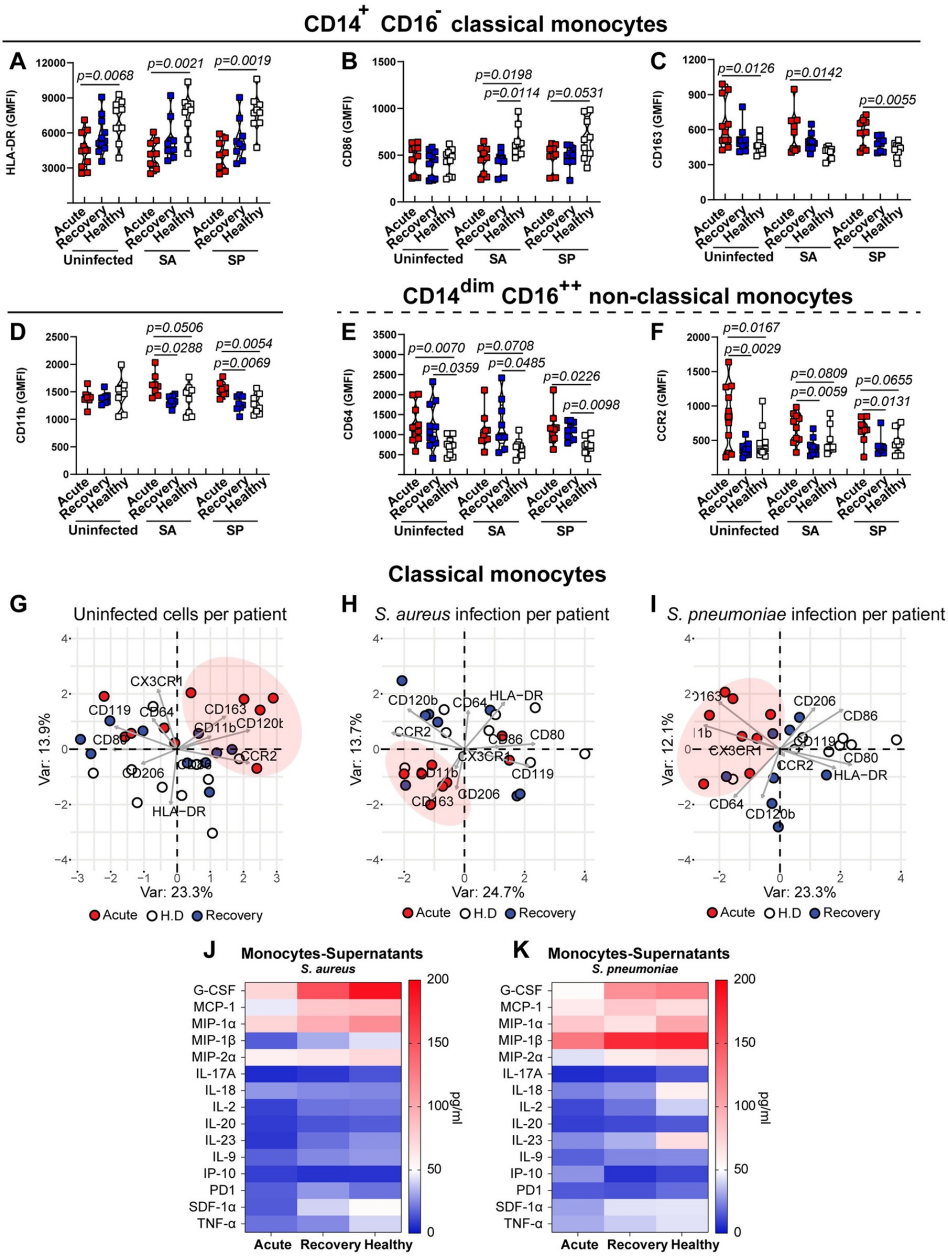
Phenotypic characterization of surface markers and secreted cytokines in monocytes upon bacterial challenge. (**A-F**) Expression of key surface markers of classical (**A-D**) and non-classical monocytes (**E and F**) from acute- or rec-phase COVID-19 patients or healthy donors pre-exposed to 10% autologous plasma for 3h and subsequently challenged with either SA or SP at MOI 1 or left unchallenged (n = 9–11). (**G-I**) PCA of cell surface phenotype of COVID-19 patients acute- (red) or rec-phase (blue) and healthy donors’ (white) classical monocytes at the basal level without bacterial challenge (**G**), upon SA infection (**H**) or SP infection (**I**). Each symbol represents cells from a single COVID-19 patient or healthy donor exposed to either COVID-19 or healthy donor plasma from one donor. (**J and K**) Heat map of cytokines secreted by monocytes from COVID-19 patients (acute-phase and rec-phase) and healthy controls after bacterial challenge with SA (**J**) or SP (**K**) (n = 2–3). For panels A-F, p values were determined by using Mann-Whitney test.

Beyond neutrophils, the monocyte compartment is particularly affected by COVID-19 [20]. Classical monocytes from acute-phase patients displayed more heterogeneity, with higher expression of CD163, CX_3_CR1 and lower expression of HLA-DR compared to recovery and healthy monocytes (Fig 6A and S6C and S8A Figs). Upon bacterial challenge, classical monocytes from COVID-19 patients (acute- and rec-phase) displayed high expression of CD163 and CD11b, but low expression of the activation markers HLA-DR, CD86 and CD80 (Fig 6A–6D and S8A Fig). Non-classical monocytes showed higher expression of CCR2, CD11b and CD163 in acute-phase, while CD64 was increased in both acute- and rec-phase compared to healthy controls, both in the presence and absence of bacterial challenge (Fig 6E–6F and S8B Fig). Overall, PCA of classical monocytes revealed an acute-phase COVID-19 clustering pattern upon bacterial challenge, which was strongly associated with low expression of HLA-DR, CD86, CD80 and a high expression of CD163, CX3CR1 and CD11b (Fig 6G–6I). Similarly, a clustering pattern of acute-phase COVID-19 non-classical monocytes upon bacterial challenge was characterized by an increased expression of CCR2, CD163 CD120b, CD11b and low expression of HLA-DR (S8C Fig). Finally, COVID-19 monocytes showed a dampened cytokine secretion profile upon bacterial challenge compared to healthy controls (Fig 6J and 6K). Particularly, monocytes from patients with acute COVID-19 secreted reduced G-CSF, MIP-1α, MIP-1β, MCP-1, TNF-α and IL-2 levels upon bacterial challenge (Fig 6J and 6K).

Taken together, these data suggest that dynamic changes of monocyte receptor and cytokine secretion profiles associated with acute COVID-19 were involved in an aberrant antibacterial response.

### Combination of TNF-α, IFN-γ and IL-4 drives impaired antibacterial effector functions of myeloid immune cells in critically ill COVID-19 patients

**Fig 7:**
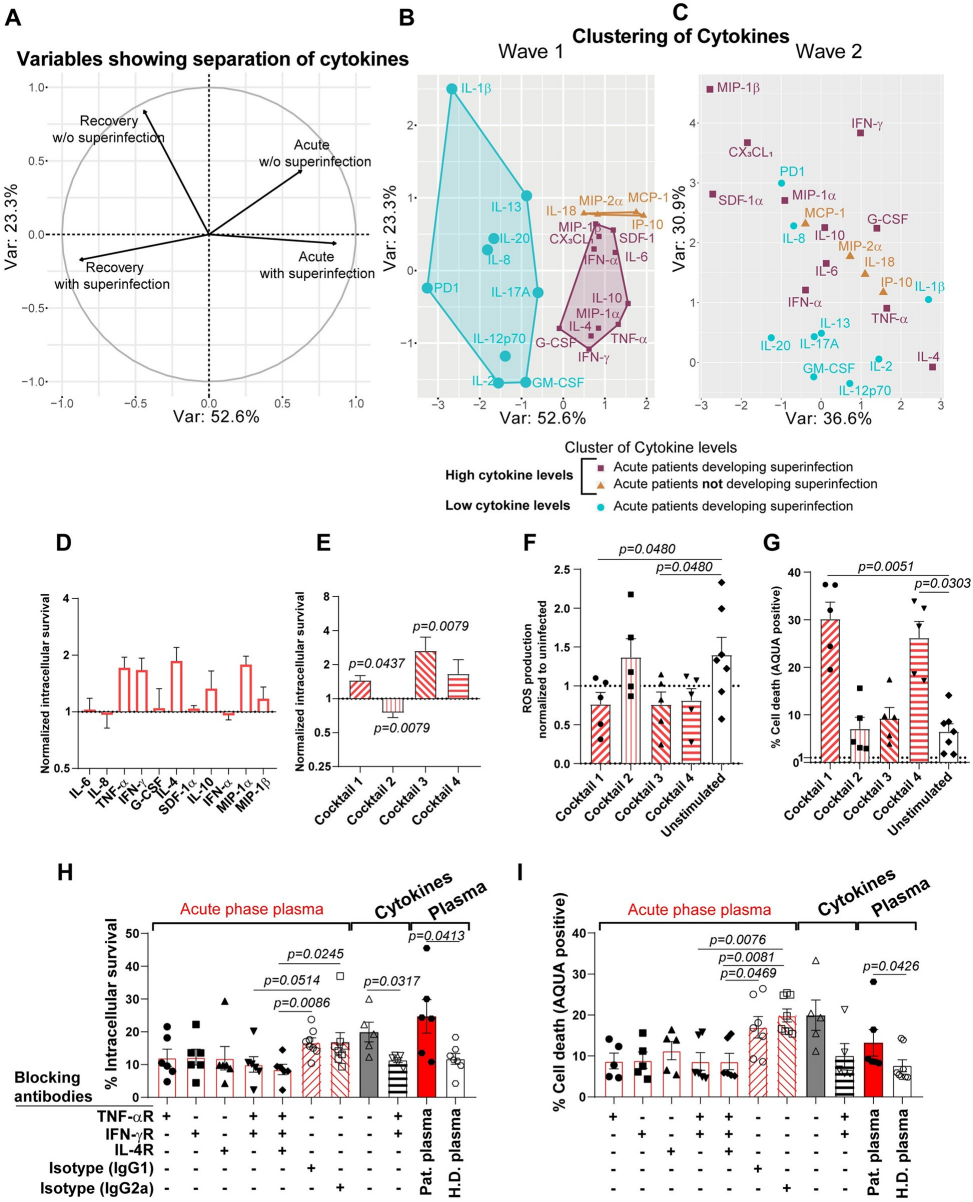
Plasma cytokine levels quantification of COVID-19 patients according to superinfection status and functional cytokine analysis. **(A)** PCA of cytokine levels in COVID-19 patients with secondary bacterial infections (superinfection) vs COVID-19 patients without superinfection, showing the separation axis between acute- and rec-phase. **(B and C)** PCA of plasma cytokine level comparison between acute- and rec-phase of wave 1 and wave 2 patients highlighting a cluster of elevated levels of cytokines in COVID-19 patients with superinfection (purple), cluster with elevated levels of cytokines in COVID-19 patients without superinfection (orange) and cluster with similarly low levels in acute- and rec-phase in patients without superinfection (cyan). Axes in both graphs are the same and based on the direction and orientation of variables as shown in panel A. Anti-inflammatory treatment received by the patients either at physician’s discretion **(B)** or following the RECOVERY trial protocol with dexamethasone **(C)**. Analysis was only done on patients not suffering from superinfection at time of sampling. (**D and E**) Intracellular killing capacity of healthy donor neutrophils (n = 5–7) pre-exposed to the indicated single (**D**) or combination of cytokines for 4 h (**E**) and subsequently infected with SA at MOI 10. Values are normalized to unstimulated neutrophils. (**F and G**) ROS production (**F**) and cell death (**G**) of healthy donor neutrophils (n = 5–7) pre-exposed to different cytokine cocktails for 4h and subsequently infected with SA at MOI 1. Cocktail 1, TNF-α and IFN-γ; cocktail 2, IL-6, IL-8, G-CSF and IFN-α; cocktail 3, IL-4, IL-10 and IFN-γ; cocktail 4, IL-4, IL-6, IL-8, IL-10, TNF-α, IFN-α, IFN-γ, G-CSF, SDF-1α, MIP-1α and MIP-1β. (**H-I**) Intracellular bacterial survival (**H**) and cell death (**I**) of healthy donor neutrophils (n = 5–8) pre-treated with receptor blockers or isotype controls and exposed to 10% acute-phase COVID-19 plasma (“Acute-phase plasma”, n = 6 plasmas). Employment of receptor blockers or isotype controls, pre-exposed to TNF-α and IFN-γ (“Cytokines”) with or without receptor blockers and 10% acute-phase COVID-19 or 10% healthy donor plasma (“Plasma”, n = 6 plasmas) for 4 h and subsequently infected with SA at MOI 10 (**H**) or 1 (**I**). Each symbol represents cells from one healthy donor. For graphs presented in (**E-G**) p values were determined by using Mann-Whitney test, (H-I) by using Kruskal-Walis test with Dunn’s multiple comparison test and Mann-Whitney test.

Based on the previous findings of altered cytokine signaling pathways, hypercytokinemia affecting neutrophils and monocytes, we were interested in determining the specific cytokines affecting the antibacterial response. As such, we compared the plasma cytokine levels of critically ill COVID-19 patients from the first wave, March to June 2020, vs the second wave, July to December 2020, and stratified by status of bacterial superinfection or not. Importantly, treatment with dexamethasone according to the RECOVERY trial was administered during the second wave, but not during the first wave [40]. PCA values of cytokines demonstrated a clear separation between patients in their acute- or rec-phase as well as for bacterial superinfection status (Fig 7A). Furthermore, a clear clustering of cytokines (IL-6, TNF-α, IFN-γ, IFN-α, SDF-1α, MIP-1α, MIP-1β, G-CSF, IL-10, CX_3_CL_1_ and IL-4) was observed in a PCA for critically ill COVID-19 patients during the first wave who presented a bacterial superinfection during their stay at the ICU (Fig 7B). These cytokines were significantly elevated in patients with bacterial superinfection during the first wave (S9 Fig). Clustering of IL-18, IP-10, MCP-1 and MIP-2α characterized patients in acute-phase COVID-19 without bacterial superinfections (Fig 7B). In contrast, a similar analysis between acute- (n = 38) and rec-phase (n = 21) second wave COVID-19 patients who were treated with dexamethasone did not reveal any specific cytokine clustering among acute-phase patients with or without bacterial superinfection (Fig 7C and S9 Fig). To investigate the potential role of this specific cytokine cluster within patients who were to develop a superinfection on the functional response capacity of neutrophils and monocytes, we performed cytokine stimulation assays with healthy donor neutrophils using either single cytokines or a combination of cytokines (cocktails) thereof prior to bacterial challenge. We discovered strong effects for five cytokines, which lead to increased intracellular bacterial survival in neutrophils (Fig 7D). This phenotype was significantly increased when the cells were stimulated with different cytokine combinations. Stimulation with cocktail 1 (TNF-α and IFN-γ) and cocktail 3 (IL-4, IL-10 and IFN-γ) increased intracellular bacterial survival, whereas stimulation with cocktail 2 (IL-6, IL-8, G-CSF and IFN-α) decreased intracellular bacterial survival (Fig 7E). Of note, stimulation with cocktail 4, containing all cytokines identified in the cluster associated with superinfections (IL-4, IL-6, , IL-10, TNF-α, IFN-α, IFN-γ, G-CSF, SDF-1α, MIP-1α and MIP-1β) except CX_3_CL_1_, but including IL-8, which was previously described as a strong predictor for COVID-19 disease severity [26], potentially by its effect on neutrophils, a trend towards increased intracellular bacterial survival was observed (Fig 7E). Furthermore, stimulation of neutrophils with cocktail 1 and 3 led to significantly decreased ROS production upon SA infection (Fig 7F), while no single cytokine stimulation led to significantly decreased ROS production (S10A Fig). Cocktail 1 and 4 led to significantly increased cell death (Fig 7G), attributable to the presence of TNF-α and IFN-γ in these cocktails, while TNF-α was the only single cytokine leading to significantly enhanced cell death of neutrophils (S10B Fig). Furthermore, TNF-α alone, cocktail 1 or cocktail 4 stimulation was sufficient to induce increased cell death by itself, without a secondary bacterial challenge (S10C and S10D Fig). We also observed a similar phenotype for monocytes, where pre-treatment with individual cytokines, especially TNF-α and CX_3_CL_1_, showed increased intracellular survival upon bacterial infection (S11A Fig). In addition, pre-stimulations with cocktail 1 (TNF-α and IFN-γ) and 3 (IL-4, IL-10 IFN-γ and CX_3_CL_1_) led to significantly higher intracellular bacterial survival, while a trend towards higher intracellular survival upon cocktail 4 treatment was observed (S11B Fig).

Finally, to confirm the importance of these identified cytokines in the observed impaired antibacterial effector function of COVID-19 myeloid immune cells, we utilized cytokine receptor blocking antibodies prior to stimulation of healthy donor neutrophils with COVID-19 patient plasma, followed by bacterial challenge. We found that blocking of the receptors for TNF-α, IFN-γ or IL-4 led to decreased intracellular bacterial survival, whereas the combination of either TNF-α and IFN-γ or TNF-α, IFN-γ and IL-4 receptor blocking led to significantly decreased intracellular bacterial survival (Fig 7H). The same combination of receptor blockers also restored neutrophil viability (Fig 7I) and ROS production upon COVID-19 plasma stimulation and subsequent bacterial challenge (S10E Fig).

Overall, these findings identified the combined effect of elevated TNF-α, IFN-γ and IL-4 plasma levels in critically ill COVID-19 patients as some of the potent drivers of decreased antibacterial effector functionality of myeloid immune cells.

### Plasma proteomics reveals reduction of inflammatory mediators in critically ill COVID-19 patients treated with dexamethasone

**Fig 8:**
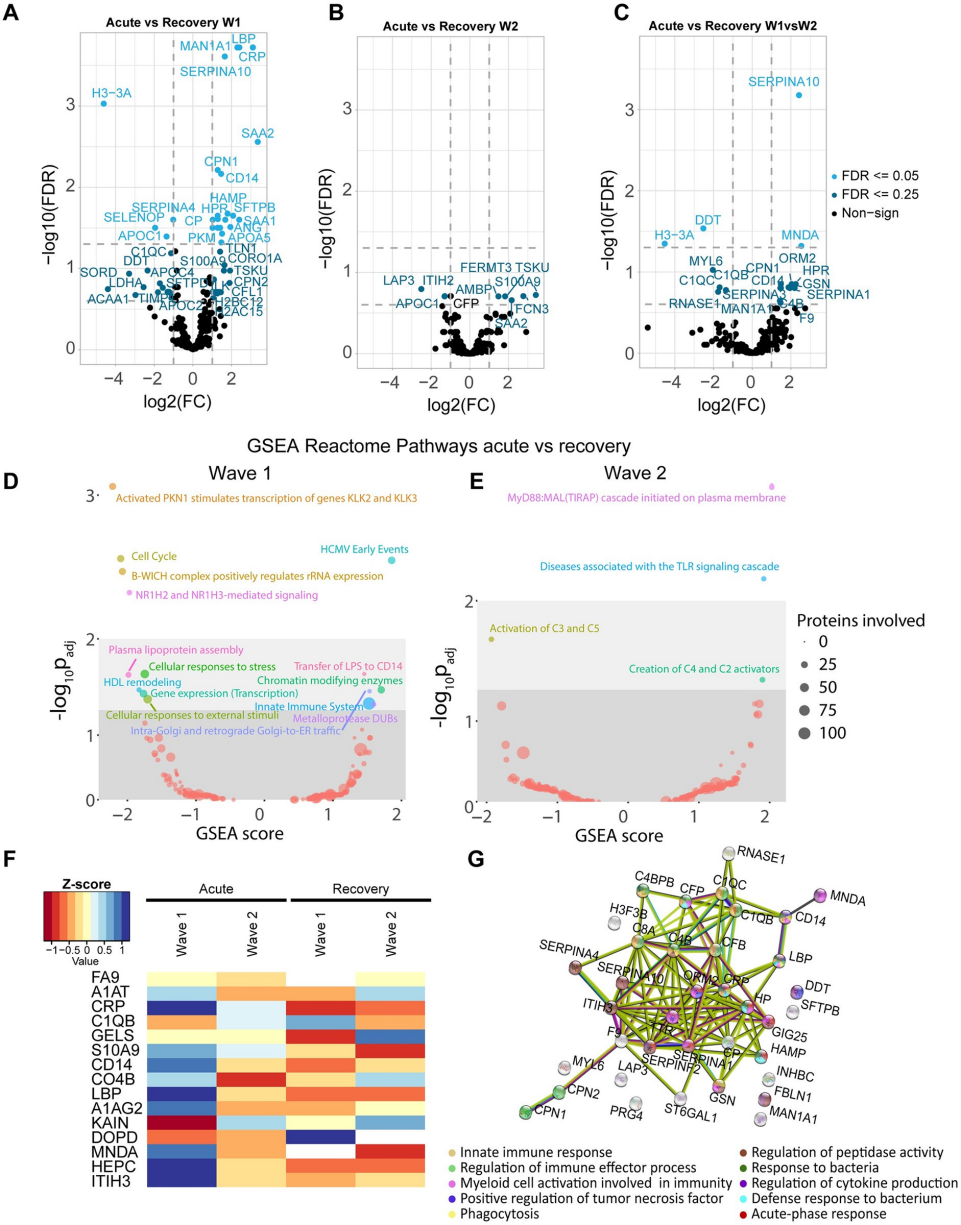
Plasma-protein profile evaluation for COVID-19 patients in acute- and their corresponding rec-phase between wave 1 and wave 2. **(A-C**) Volcano plots depicting the changes in plasma protein intensities based on the patient condition (acute- vs rec-phase) in COVID-19 wave 1 (**A**), wave 2 (**B**) or between acute- vs rec-phase in wave 1 compared to acute- vs rec-phase in wave 2 (**C**). A minimum change of two folds and an FDR of 0.05 and 0.25 was considered for statistical significance. (**D-E**) Gene set enrichment analysis (GSEA) was done to depict changes in the expression of proteins involved in different cellular pathways using the Reactome across conditions (acute-phase vs rec-phase) in COVID-19 wave 1 (**D**) and wave 2 (**E**). The diameter of the circles depicts the number of proteins involved in the illustrated process and the color indicate a specific pathway. (**F**) A heatmap of the Z score of signal intensities depicting the changes of the top 15 proteins measured in the plasma samples and with a known role in immune response to infections across conditions (acute-phase vs rec-phase) from COVID-19 patients during wave 1 and wave 2. (**G**) An interaction map was constructed to depict the signal intensity changes of proteins between acute- vs rec-phase in wave 1 compared to acute- vs rec-phase in wave 2 using the STRING database. The color of the spheres illustrates the functional group they belong to, while the color of the connecting strings shows the type of interaction as given by STRING.

Beyond the analysis of the plasma cytokine levels in COVID-19 acute- and rec-phase, we sought to determine whether any other soluble proteins in the plasma could be a driver behind the impaired antibacterial effector response of myeloid immune cells. We performed an evaluation of the protein changes in the plasma of these patients via TIMS-TOF-MS. We identified a total of 308 proteins in 33 plasma samples, including 16 patient pairs (acute- and rec-phase) of which eight were from critically ill COVID-19 patients from each wave 1 (without dexamethasone treatment) and wave 2 (with dexamethasone treatment), plus one unpaired acute-phase patient sample from wave 1. The wave 1 acute-phase patients samples presented a clear clustering and separation from the wave 2 plasma samples in a PCA analysis according to changes in their plasma protein profile (S12A Fig). We observed a significant increase in the number of proteins with changes in their intensity between acute- and rec-phase plasma from wave 1 (49 proteins) as compared to wave 2 (9 proteins) (S4 and S5 Tables). A volcano plot of the signal intensities showed a more than two-fold increase in proteins involved in tissue protection, such as SERPINA10, the inflammatory markers CRP, LBP, TSKU, SAA2 and S100A9 as well as the myeloid immune cell marker CD14 in wave 1 during their acute-phase as compared to their rec-phase (Fig 8A and S4 Table). For wave 2, differences in protein intensities were only observed with an FDR of 0.25 and interestingly also included higher levels of SAA2, S100A9 albeit with a much lower difference in intensity between acute- and rec-phase as compared to wave 1 (Fig 8B and S5 Table). Additionally, the evaluation of significant intensity changes between proteins in wave 1 and wave 2 patient according to their condition (acute- vs rec-phase) showed a significantly higher fold change in wave 1 as compared to wave 2 with proteins such as CD14, HPR, MNDA and several SERPINAs (Fig 8C and S6 Table), indicating a significant difference in the levels of inflammatory mediators between wave 1 and wave 2. Whereas proteins, such as RNASE1, DDT, H3-3A and complement factors, were at a lower level in wave 1 as compared to wave 2 (Fig 8C). As for the different pathways affected in patients from both waves, we observed a significant dysregulation of several metabolic processes by Reactome (Reactome database) gene set enrichment analysis (GSEA) in acute-phase compared to their rec-phase between wave 1 and 2 (Fig 8D and 8E), especially in innate immune defense and cellular response to stress. A significantly higher variation was observed in the number of proteins involved in immune defense and metabolic processes for wave 1 as compared to wave 2 (S12B Fig). We also analyzed all the proteins with significant changes by normalization with Z-score and generated a heat map, revealing that inflammatory markers such as CRP, S100A9, CD14, LBP and MNDA had a higher intensity in acute patients from wave 1 than wave 2 (Fig 8F and S12C Fig). Similarly, proteins such as KAIN (SERPINA4) and DOPD (DDT) had lower intensity in acute-phase patients from wave 1 than wave 2 (Fig 8F and S12C Fig). Finally, a STRING interaction analysis was performed to identify enriched pathways and the roles, as well as interactions, of proteins with significant differences between acute- and rec-phase of wave 1 patients compared to the differences between acute- and rec-phase of wave 2 patients. A high number of the identified proteins were strongly linked together due to their involvement in innate immune response, defense response to bacteria, cytokine production and myeloid cell activation as well as in regulation of the immune effector response towards infections (Fig 8G).

These results deliver important insights into further potential drivers underlying the observed impaired antibacterial effector response of myeloid immune cells of critically ill COVID-19 patients beyond cytokine-mediated effects by means of complex inflammatory protein interactions. Furthermore, they also emphasize the potentially beneficial effects of broad anti-inflammatory treatment in these patients by dampening the complex inflammatory environment, aiming at restoring homeostasis.

### Improved myeloid cell effector response against bacterial challenge and decreased levels of inflammatory mediators are associated with dexamethasone treatment

**Fig 9:**
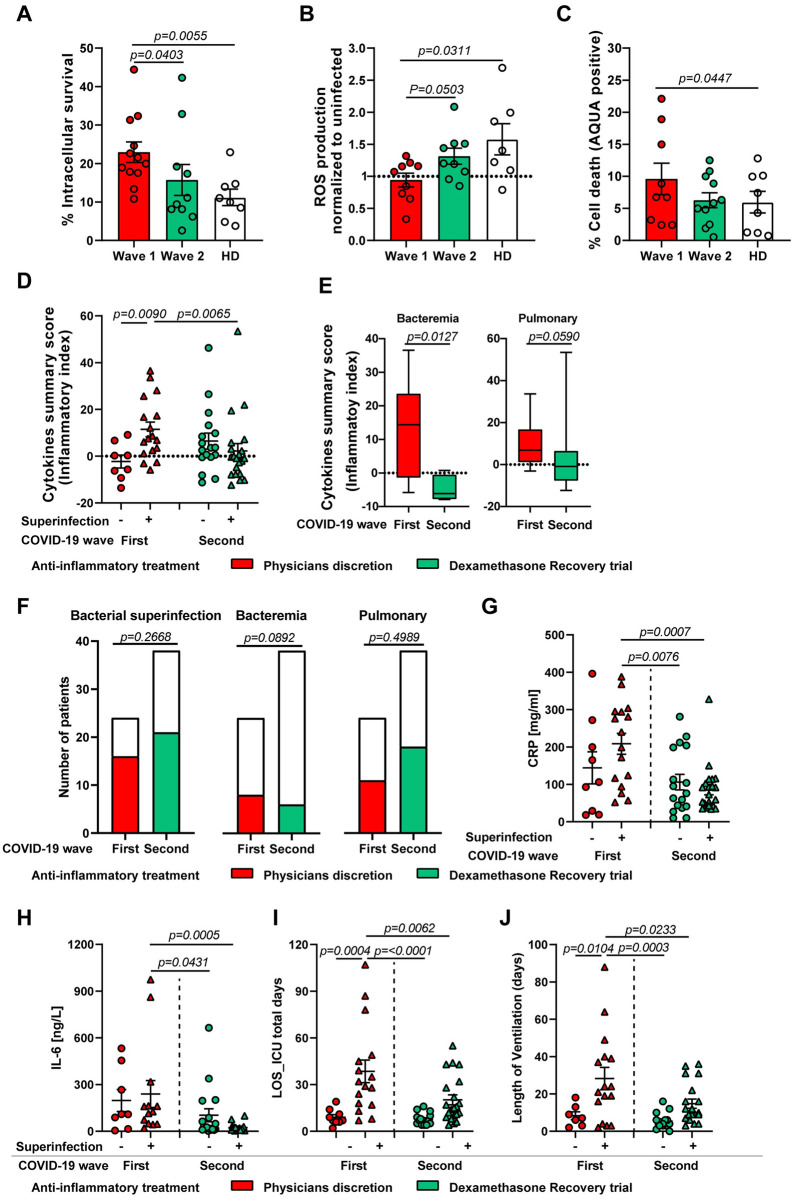
Quantification of inflammatory mediators in plasma and its association with bacterial superinfection compared between the first and second COVID-19 wave. (**A-C**) Intracellular bacterial survival (**A**), ROS production (**B**) and cell death (**C**) of healthy donor neutrophils (n = 6) pre-exposed to either 10% of wave 1 acute-phase (n = 9–12), wave 2 acute-phase (n = 8–10) or healthy donor plasma (n = 7–8) for 3 h and subsequently infected with SA at MOI 10 (**A**) or 1 (**B and C**). (**D and E**). Normalized cytokine values (sum of Z-scores) depicted as cytokine summary score (inflammatory index) in the plasma of acute-phase COVID-19 patients between first and second wave with or without superinfection. The cytokine summary score from first wave is also presented in Fig 1D (**D**) and stratification by type of superinfection, i.e. bacteraemia (left) or pulmonary infection (right) between first wave (red box) and second wave (green box) (**E**). Data presented as whisker plots with box indicating interquartile range and error bars indicating highest and lowest value. (**F and G**) Contingency graph showing the difference in proportion of total bacterial superinfection, bacteraemia status and pulmonary infection status (**F**) in critically ill COVID-19 patients during first wave and the second wave. (**G-J**) Clinical as well as laboratory parameters that are part of the routine diagnostics including, CRP (**G**) and IL-6 levels (**H**) as well as length of ICU stay (**I**) and ventilation (**J**) of acute-phase COVID-19 patients between first and second wave with or without superinfection. For panels **A-C**, each symbol represents cells from one healthy donor treated with 10% plasma, for panels **D, G-J**, each symbol represents one human subject. For panels **A-E** and **G-J**, p values were determined by using Mann-Whitney test, for panel **F** by using Chi-square test.

We hypothesised that the dampened inflammatory plasma environment of COVID-19 patients treated with dexamethasone in wave 2 might also improve the antibacterial effector response of myeloid immune cells. Therefore, we carried out stimulation assays using healthy donor neutrophils or monocytes with plasma from either wave 1 or wave 2 patients and assessed their effector response towards bacterial challenge. We observed significantly increased intracellular bacterial survival (Fig 9A), decreased ROS levels (Fig 9B) and enhanced cell death (Fig 9C) in neutrophils stimulated with wave 1 plasma as compared to healthy plasma stimulation, while no such differences were observed upon wave 2 plasma stimulation. There was no difference in phagocytosis upon plasma only stimulation by either wave 1 or wave 2 plasma (S13A Fig). There was a trend towards increased background cell death upon plasma only stimulation by wave 1 as compared to wave 2 plasma (S13B Fig). Similarly, we also observed increased intracellular bacterial survival in monocytes (S13C Fig), decreased ROS production (S13D Fig) and increased cell death (S13E Fig) of monocytes stimulated with wave 1 plasma as compared to healthy plasma, but not upon wave 2 plasma stimulation upon bacterial infection. While no differences in HLA-DR expression were identified upon bacterial infection or upon only plasma stimulation (S13F and S13G Fig). However, monocytes exhibited increased background cell death upon plasma only stimulation by wave 1 as compared to wave 2 plasma (S13H Fig).

Based on these experimental findings and the prior identification of the hyperinflammatory environment including cytokines as driver of the observed phenotype, we reasoned that targeting and thereby decreasing elevated levels of soluble inflammatory mediators might contribute to a reduction of bacterial superinfections and thus improve patient outcome. An analysis of the first wave of COVID-19 patients who have developed a bacterial superinfection showed significantly elevated cumulative cytokine levels as measured by cytokine summary score (inflammatory index) in both acute- and rec-phase (Figs 1D and 9D and S14A Fig). Whereas no differences in cytokine summary score were detected between patients with or without superinfection in both acute- and rec-phase in the second wave (Fig 9D and S14A Fig). Similarly, the cytokine summary score of wave 1 patients with superinfection was significantly higher than the cytokine summary score of wave 2 patients with superinfection (Fig 9D and S14A Fig). Additionally, the cytokine summary score in plasma from patients who suffered from bacteraemia was significantly reduced in the second wave as compared to the first wave (Fig 9E, left) and showed a tendency towards decreased levels in patients with pulmonary superinfection (Fig 9E, right). However, there was no difference in the proportion of total bacterial superinfection cases or pulmonary superinfections between wave 1 and wave 2 (Fig 9F). A lower proportion of bacteraemia cases, although not statistically significant, was identified in the second wave as compared to the first wave (Fig 9F).

Furthermore, a comparison of the levels of important inflammatory and clinical markers in COVID-19 patients between the first and second wave showed a significant reduction of C-reactive protein (CRP) (Fig 9G) and IL-6 levels (Fig 9H) as well as decreased length of ICU stay (Fig 9I) and ventilation days (Fig 9J) in patients suffering from a bacterial superinfection during the second wave. Of note, no differences in other clinical and laboratory parameters including neutrophils, monocytes or leukocytes counts, SAPS, SOFA score, age, LDH, D-dimer levels and time to superinfection were observed between the first and second wave of COVID-19 patients (S14B–S14K Fig). These results suggest a potential role for the association of elevated inflammatory mediators found in the plasma of critically ill COVID-19 patients and increased risk for developing bacterial superinfections. Furthermore, dampening the excessive inflammatory environment can improve the antibacterial effector functionality of neutrophils and monocytes.

## Discussion

In this study, we show that a higher degree of SARS-CoV-2 mediated hypercytokinemia in the plasma is positively associated with bacterial superinfections. By assessing key functional and phenotypic properties of neutrophils and monocytes from acute-phase COVID-19 patients, we show that these cells exhibit multiple functional changes, including impaired microbicidal capacity, reflected by abrogated ROS and MPO production as well as NETs formation by neutrophils and impaired ROS production in monocytes upon bacterial challenge. This impaired phenotype was characterized by a high expression of CD66b, CXCR4 and low expression of CXCR1, CXCR2 and CD15 in neutrophils and low expression of HLA-DR, CD86 and high expression of CD163 and CD11b in monocytes. In addition, we also addressed the role of COVID-19 superinfection-associated cytokines such as TNF-α, IFN-γ and IL-4, which synergistically mediate impaired antibacterial effector function in myeloid immune cells. Furthermore, anti-inflammatory treatment with the corticosteroid-dexamethasone was associated with enhanced cellular effector response towards bacterial challenge, reduction of inflammatory mediators in the plasma as well as the reduction in CRP, IL-6 levels, length of ICU stay and ventilation-days in critically ill COVID-19 patients.

Studies have shown that severe COVID-19 is accompanied by hypercytokinemia with high levels of pro-inflammatory cytokines such as IL-6 and IL-1β as well as anti-inflammatory cytokines such as IL-4 and IL-10 [12,14,41]. Our initial Luminex-based screening on plasma from critically ill COVID-19 patients from the first wave detected significantly higher levels of cytokines involved in recruitment and trafficking of neutrophils such as IL-8, G-CSF and SDF-1α, in accordance with previous reports showing that acute-phase COVID-19 patients have elevated neutrophil counts [6,20,22,42,43]. We also report an increase in CX3CL1, IP10 and MIP-1β levels, indicating increased recruitment of monocytes [12,43]. However, the concomitant presence of high levels of IL-4 and IL-10, with broad anti-inflammatory functions, might cause functional impairment of neutrophils and monocytes towards bacterial challenge [44].

Indeed, we observed that neutrophils and monocytes derived from acute-phase COVID-19 patients showed a decreased capacity to kill intracellular bacteria, which was linked to a significant decrease in their ability to produce ROS and intracellular MPO (for neutrophils).

This altered functionality is consistent with a recent study showing reduced oxidative burst of neutrophils in response to *E*. *coli* in severe COVID-19 patients [20]. Since neutrophils and monocytes engage in a complex crosstalk with other immune cells to elicit an efficient effector-response, we were keen on identifying possible autocrine-paracrine signaling mechanisms. Neutrophils and monocytes from critically ill COVID-19 patients were functionally impaired in their capacity to produce cytokines important for activation and subsequent antimicrobial actions. Significantly lower levels of G-CSF and IL-17 as well as IL-18 in neutrophils from acute patients could be linked to decreased ROS [45,46] and MPO [47] production, respectively. This was consistent with a recent observation regarding the diminished or inexistent expression of cytokine genes (*il6* and *tnf*) by monocytes upon stimulation with TLR ligands [14].

Several recent studies have proposed that NETs can contribute to inflammation-associated lung damage and microthrombi in severe COVID-19 patients [37,48]. The concentration of NETs components was found to be augmented in plasma, tracheal aspirate and lung autopsy tissues from COVID-19 patients [38,49]. Notably, it was found that SARS-CoV-2 infection could directly induce the release of NETs by healthy neutrophils [49]. In line with these findings, we observed that neutrophils from acute patients released higher levels of DNA upon COVID-19 plasma stimulation. However, the release of classical NETs against SA was significantly reduced in the acute COVID-19 environment. This phenomenon might be due to neutrophil exhaustion and a subsequent inability to properly respond to bacterial challenges.

These findings were confirmed by the fact that neutrophils isolated from acute patients showed lower expression of key receptors CXCR1, CXCR2 as well as CXCR3, important for sensing IL-8 as well as G-CSF [50,51], thereby pointing towards increased engagement of these receptors. On the other hand, SDF-1α receptor CXCR4, involved in neutrophil trafficking from the bone marrow and local retention, was significantly higher, pointing to decreased signaling engagement, whereas the maturation marker CD15 was significantly lower in acute COVID-19 cells. CXCR4 has been described as a neutrophil precursor marker [52] and similarly it has been suggested that these immature neutrophils are being released into the blood during severe COVID-19 [22]. Also, presence of abnormal neutrophils in patients with severe COVID-19 has been observed [22,53]. A recent single-cell transcriptomic study proposed that premature neutrophils in severe COVID-19 might be programmed towards an anti-inflammatory state or even exert suppressive functions, similar to myeloid-derived suppressor cells (MDSCs) [20]. Furthermore, we recently demonstrated that a subpopulation of neutrophils in critically ill COVID-19 patients undergoes TNF-α-driven necroptosis [54], with concomitant release of various damage associated molecular patterns and alarmins, such as S100A8/A9, which are known to contribute to pathological damage [22,55,56].

Acute-phase patients showed a lower proportion of classical monocytes, crucial for anti-bacterial response, compared to rec-phase patients and healthy donors. Additionally, monocytes were also characterized by lower numbers of non-classical monocytes that are important for maintaining vascular homeostasis [11,20,22,53,57,58]. Similar to other studies, HLA-DR expression on classical monocytes was significantly reduced [11,20,22], which can be mediated by IL-6 overproduction during severe COVID-19 [11]. Emergence of HLA-DR^low^ monocytes during severe COVID-19 can be linked to a phenotype similar to MDSCs or dysfunctional monocytes. HLA-DR^low^, CD163^high^ monocytes are usually associated with anti-inflammatory tissue-homeostatic functions and are linked to an immunosuppressive phenotype in sepsis [59–62]. Thus, a defective or suppressed monocyte compartment can further add to its inability to respond to bacterial infection.

We found that higher degree of plasma hypercytokinemia in COVID-19 patients correlated with the occurrence of bacterial superinfections during the first wave [31]. We detected a specific cluster of the cytokines IL-6, TNF-α, IFN-γ, SDF-1α, MIP-1α, MIP-1β, G-CSF, IL-10, CX_3_CL_1_, and IL-4, which were elevated in patients developing bacterial superinfection. Especially, TNF-α, IFN-γ and IL-4 stimulations contributed towards the observed impaired functional response along with increased cell death. In line with these findings, our receptor blocking assays targeting TNF-α, IFN-γ and IL-4 receptors on neutrophils in the COVID-19 plasma environment confirmed their major role in the observed impaired antibacterial phenotype. In a recent study, Karki *et al*. showed that TNF-α and IFN-γ synergize to induce inflammatory cell death and this synergy is linked to mortality during SARS-CoV-2 infection [18]. Similarly, IL-4-mediated receptor signaling in neutrophils is known to affect their effector response, specifically abrogating classical NETs formation [63]. Additionally, a recent study demonstrated that basophil-derived IL-4 promoted *S*. *aureus* skin infection and IL-4 receptor blocking promoted enhanced bacterial clearance in a mouse model [64]. The degree of hypercytokinemia in COVID-19 patients from the second wave, which had been treated with the corticosteroid dexamethasone, did not reveal any specific clustering of cytokines and was not associated with impaired effector response against bacterial challenge.

Moreover, we also observed a change in the proteomic profile of acute-phase patients between the first and second wave. The levels of inflammatory markers, like SENELOP, SERPINAs, LBP, and SAA2, were found to be significantly altered in acute-phase patients of both wave 1 and wave 2, respective to their rec-phase, albeit a higher alteration was found in wave 1. SAAs and SERPINAs are important for proper neutrophil functionality [56,65–67], which might play an additional role in the impairment we observed. Moreover, recent reports linked plasma proteomic changes of COVID-19 patients to different degrees of disease severity, with special emphasis on tissue repair and coagulation proteins, such as the SERPINAs, inflammatory markers and regulators, like CRP and TSKU, macrophage trafficking markers, like SELENOP, as well as immune cell markers such as LBP and CD14 [68–75]. These findings in our cohort further strengthen our hypothesis on myeloid cell dysregulation and it is in line with the reported COVID-19 proteomics data [71,74,75].

Even though corticosteroid treatment was widely used during previous viral pandemics, such as influenza [76–78], SARS-CoV [79,80] and MERS-CoV [81], its use remains controversial [82], since observational studies reported increased mortality and nosocomial infections during influenza [83,84], and delayed clearance of SARS-CoV and MERS-CoV [85,86]. So far, only a limited number of published studies on the mechanistic effects of corticosteroid therapy in COVID-19 is available [87]. However, a few studies reported improved clinical symptoms and oxygenation, reduced length of hospitalization and ICU stay as well as lower 28-day mortality among patients with invasive mechanical ventilation [6,32,40,88–90]. Hence, for critically ill COVID-19 patients with dysregulated inflammation and hypercytokinemia causing excessive tissue injury, appropriate use of corticosteroids could be beneficial [82,91]. As corroborated by our results, the highly inflammatory COVID-19 environment can cause exhaustion of innate immune function against secondary bacterial infections. Hence, targeting these inflammatory mediators by short-term usage of broad anti-inflammatory therapy, such as corticosteroids, might restore the effector function of myeloid immune cells in the host, thereby decreasing the risk of secondary bacterial infections in COVID-19.

However, we acknowledge several limitations of our study. First, the small patient sample size does not allow for further in-depth analysis of the clinical outcome. Second, our observations are limited to a single center study, hence further prospective multi-center studies with larger patient samples specifically looking at the correlation between elevated levels of certain cytokines as well as the potential beneficial role of corticosteroid treatment and the risk for developing bacterial superinfections are required to elaborate and verify the observations from our study. Third, in a recent study it was shown that dexamethasone treatment in COVID-19 patients affected circulating neutrophils, altered their IFN signaling and expanded immature neutrophils [87]. Similarly, increase in abnormal/immature neutrophils in the blood stream during severe COVID-19 has been observed by previous studies [20,22,27,53]. Whether the hyperinflammatory environment as well as dexamethasone treatment specifically effects the functional responses of these immature neutrophils against bacterial infection is largely unknown. In our study we have not investigated the role of the maturity status of the neutrophils and monocytes and their effect on antibacterial response. Hence, future studies specifically looking at the maturity and sub-type status of myeloid immune cells and their effector response against secondary bacterial infections are required.

In conclusion, we demonstrated that during acute COVID-19, critically ill patients presented with alterations in neutrophil and monocyte effector cytokines, which severely affected their ability to respond to bacterial challenges, as determined by in-depth functional and phenotypic assessment of neutrophils and monocytes. These data corroborated the clinical disease course with increased bacterial superinfections observed in critically ill COVID-19 patients in wave 1 as compared to wave 2. Our study further emphasizes the importance of tailoring treatments, aiming at restoring the antibacterial effector functions of neutrophils and monocytes, thereby decreasing the risk of elevated mortality in COVID-19 due to secondary bacterial infections.

## Materials and methods

### Ethical statement

Patients recruited under the Microbiota-COVID prospective cohort study conducted at the Institute of Intensive Care Medicine of the University Hospital Zurich (Zurich, Switzerland) registered at clinicaltrials.gov (ClinicalTrials.gov Identifier: NCT04410263). The study was approved by the local ethics committee of the Canton of Zurich, Switzerland (Kantonale Ethikkommission Zurich BASEC ID 2020–00646). Blood sampling for isolation of neutrophils, monocytes and plasma collection from healthy volunteers was approved by the local ethics committee of the Canton of Zurich, Switzerland (Kantonale Ethikkommission Zurich BASEC ID 2019–01735). Formal written consent was obtained from all the participants of the study. Patients were considered to be in the acute phase on the first 5 days after initial ICU admission while the rec-phase was defined as patients were discharged from the ICU or were negative for COVID-19 and under a defined clinical score, in a non-critical state. A list of all patient demographics and clinical scores is available in S1 and S2 Tables.

Bacterial superinfection was defined according to the previously published study by *Buehler et al*., 2021 [31]. “A multidisciplinary panel of ICU and infectious diseases consultants (unrelated to the study group) assessed the clinical status of the patients on a daily basis. Superinfection was diagnosed according to the panel’s judgement of clinical deterioration and routine laboratory assessment as well as microbiological results. In more detail, the isolation of microorganisms from respiratory specimen cultures (TBS and/or BAL) regarded as clinically relevant by the panel was used as antimicrobial treatment guidance and the first specimen without pathogen growth was considered as the end of an episode in concordance with the clinical course” [31]. Species identified causing bacterial superinfections among critically ill COVID-19 patients during wave 1 and wave 2 are depicted in (S15A and S15B Fig).

### Bacterial strains

*Staphylococcus aureus* (SA) strains JE2 (MRSA-USA300, NARSA) and Cowan I (MSSA, ATCC 12598) were grown in Tryptic Soy Broth (TSB) at 37°C and 220 rpm for 16 h. Stationary phase cultures were diluted in fresh TSB and bacteria were grown to exponential phase for the infection. For *Streptococcus pneumoniae* (SP), the 603 strain (serotype 6B) [92] was passaged twice on blood agar plates (Columbia blood agar, Biomereux) and incubated at 37°C with 5% CO_2_ for 14 h. A liquid culture was prepared in Todd Hewitt Yeast broth (THY) with a starting OD_600nm_ of 0.1 and grown at 37°C in a water bath until OD_600nm_ of 0.35 for the infection.

### Blood collection and plasma preparation

COVID-19 patients and healthy donors’ blood was sampled in EDTA tubes containing 3x10 ml of blood from each patients as per the protocol under the Microbiota-COVID (ClinicalTrials.gov Identifier: NCT04410263), (BASEC ID 2020–00646) and centrifuged at 1800g for 10 min for plasma collection. The collected plasma was centrifuged a second time at same conditions to remove any additional debris and supernatants were collected and aliquoted. Fresh plasma was immediately used to prepare a 10% plasma solution in RPMI 1640 (Gibco, ThermoFisher) and used for *in vitro* experiments; the remaining plasma was utilized for cell stimulation as well as cytokine quantification and remaining aliquots stored at -80°C until further use.

### Peripheral blood mononuclear cells (PBMCs) isolation

Patients and healthy donor PBMCs were isolated from the cellular fraction of the blood after 1:2 dilution with DPBS using the Lymphoprep (Axis Shield) density gradient method. In brief, the diluted blood was overlaid on Lymphoprep and centrifuged for 25 min at 840g with lowest acceleration and break settings. Following the gradient separation, the PBMCs layer was transferred into a new 50 ml conical tube and diluted with FACS buffer (2 mM EDTA and 1% FBS). Cells were washed twice with FACS buffer. Next, cells were resuspended in red blood cells (RBC) lysis buffer (ThermoFisher), mixed gently and incubated for 10 min at 37°C and 5% CO_2_. The lysis reaction was stopped by adding FACS buffer and the suspension was centrifuged. Cells were washed once and resuspended in FACS buffer for counting using the Attune NxT flow cytometer (ThermoFisher).

### Monocytes enrichment from PBMCs

Patients and healthy PBMCs were used for monocyte enrichment using the EasySep Human Monocyte Enrichment Kit without CD16 Depletion (StemCell technologies) following the manufacturer’s instructions. In brief, PBMCs (<100 million) were transferred to a 5 ml polystyrene tube, the human monocyte enrichment cocktail was added and the sample was gently mixed and incubated for 10 min on ice. Following incubation, magnetic beads were added to the mixture and samples were mixed and incubated for 10 min on ice. Finally, the mixture was placed in a magnetic holder (StemCell technologies) for 3 min and the cells were decanted into a new tube. Monocytes were then washed, resuspended in RPMI 1640 and counted using the Attune NxT flow-cytometer (ThermoFisher).

### Neutrophil isolation

Neutrophils were isolated from the cellular fraction of the blood, after dilution with DPBS (Gibco, ThermoFisher), with the EasySep Human Neutrophil Isolation Kit (StemCell technologies) according to the manufacturer’s instruction. In brief, Neutrophil enrichment cocktail was added to the diluted blood and incubated for 15 min at RT. Next, magnetic beads were added for another 15 min, after which the tubes were placed into a magnetic holder (StemCell technologies). PMNs were collected after 15 min of cell separation. They were centrifuged at 470g for 6 min (low acceleration and brakes) and subsequent red blood cells (RBCs) lysis was performed with resuspension in H_2_O followed by addition of DPBS. After a further centrifugation step, neutrophils were resuspended in RPMI 1640 and counted using the Attune NxT flow-cytometer (ThermoFisher).

### Cytokine measurement by Luminex

Cytokine levels in patients and healthy donors’ plasma, as well as cell culture supernatants from *ex vivo* experiments were assessed using the Luminex MAGPIX instrument (ThermoFisher). Samples were thawed on ice and prepared according to the manufacturer’s instructions using a custom-made 33-plex human cytokine panel (Procartaplex ThermoFisher). In brief, Luminex magnetic beads were added to the 96-well plate placed on a magnetic holder and incubated for 2min. The plate was washed twice with assay buffer for 30 sec. In parallel, provided standards and plasma samples were diluted in assay buffer (cell culture media was used for cell culture supernatants) and added to the plate. The plate was incubated for 2h at RT at 550 rpm in a plate orbital shaker. Next, the plate was washed twice with assay buffer and incubated for 30 min at 550 rpm with detection antibodies. After two washing steps, the plate was incubated with Streptavidin-PE solution for 30 min at 550 rpm. Finally, the plate was washed, reading buffer was added and incubated for 10 min at RT and 550 rpm before running the plate. Data acquisition and analysis were performed using the Xponent software (v. 4.3). Data were validated using the Procarta plex analyst software (ThermoFisher).

### Principal component and integrated correlation analysis

PCA plots of the cytokine analysis from patient and healthy donors’ plasma as well as receptor analysis from the *ex vivo* experiments were created using the ‘PCA’ and the ‘fviz_pca_biplot’ functions available in ‘FactoMineR’ package in R. Correlation mapping was performed using the ‘corrplot’ package in R. The color of the circles indicated positive (blue) and negative (red) correlations, color intensity represented correlation strength as measured by the Pearson’s correlation coefficient. The correlation matrix was reordered manually to better visualize the variables of interest.

### Plasma stimulation

Isolated neutrophils or monocytes from both COVID-19 patients and healthy donors were seeded in 96-well V-bottom plates (for flow cytometry assays, around 2x10^5^ cells/well) or in 24-well F-bottom plates (for phagocytosis and intracellular survival assays, approximately 2.5x10^5^ to 3x10^5^ cells/well). Initially a titration experiment was performed to determine the plasma concentrations required for further stimulations and functional assays. Neutrophils from healthy donors were stimulated with 1%, 5%, 10% and 20% concentrations of acute-phase plasma (S15C–S15E Fig). Based on these data myeloid immune cells were stimulated with 10% autologous or heterologous (either COVID-19 patients or healthy donor) plasma for 3 h at 37°C + 5% CO_2_. Followed by bacterial challenge for defined time points based on assay requirement (see below). Additionally, confirmatory experiments including healthy donor monocytes derived from buffy coats were stimulated with 10% autologous or heterologous (either COVID-19 patients or healthy donor) plasma for overnight at 37°C + 5% CO_2_.

### Cytokine stimulation and receptor blocking assay

Healthy neutrophils and monocytes were seeded in 96-well V-bottom plates (Flow cytometry assays, with 2x10^5^ cells/well) and in 48-well plates (phagocytosis and intracellular survival assays at a concentration of 2.5x10^5^ to 4x10^5^ cells/well). Cells were stimulated with different cytokines (alone or in combination as a cocktail) in RPMI for 4 hours (neutrophils) and overnight (monocytes) at 37°C and 5% CO_2_ at a 20 ng/ml concentration for IL-6, IL-8, IL-4, TNF-α, IFN-α, IFN-γ, G-CSF, IL-10, MIP-1α, MIP1-β, and CX_3_CL_1_ as well as SDF-1α at 35 ng/ml concentration were used to stimulate both neutrophils and monocytes. CX_3_CL_1_ was used for monocytes only. COVID-19 patients or healthy donors’ plasma (10%) was used as control. Additionally, combinations of cytokines were utilized for the stimulations termed as cocktails. Cocktail 1 (TNF-α and IFN-γ, due to their cell death mediating properties), cocktail 2 (IL-6, IL-8, G-CSF and IFN-α as a pro-inflammatory mediator), cocktail 3 for neutrophils (IL-4, IL-10 and IFN-γ) or for monocytes (IL-4, IL-10, IFN-γ and CX_3_CL_1_) as anti-inflammatory/chemotactic and homeostasis mediator and cocktail 4, containing all cytokines identified in the cluster associated with superinfections (IL-4, IL-6, , IL-10, TNF-α, IFN-α, IFN-γ, G-CSF, SDF-1α, MIP-1α, CX_3_CL_1_, MIP-1β) and IL-8 mimicking COVID-19 associated hypercytokinemia.

For the receptor blocking assay, healthy neutrophils were isolated and seeded as described previously. Next, single or a cocktail of specific antibodies against IFNGR1 (1 μg/ml, MAB6731-SP, R&D systems), TNF R1 (1 μg/ml, 55R-170, ThermoFisher) and IL4 Rα (1 μg/ml, MAB230-100, R&D systems) were added to the cells in RPMI. IgG1 (Clone # 11711, R&D systems) and IgG2a (clone Clone # 20102, R&D systems) isotype antibodies were used as controls. The neutrophils were preincubated with the blocking antibodies for 30–60 min at 37°C and 5% CO_2_ after which 10% patient plasma was added and the cells were further incubated for 3 h. COVID-19 patients or healthy donor plasma (10%) was used as experimental control for cells without any additional stimulation.

### Bacterial challenge

For phagocytosis and intracellular killing assays, bacteria were opsonized for 20 min in RPMI 1640 supplemented with 2.5% of either patient or healthy donors’ plasma at a determined multiplicity of infection (MOI) for neutrophils (50 for SP and 10 for JE2-SA) and monocytes (50 for SP and Cowan I-SA) infections respectively.

To analyze intracellular survival of the bacteria in neutrophils, isolated neutrophils were seeded into 24-well plates (TPP), pre-stimulated with 10% plasma (see above) and infected with exponentially grown SA at a MOI of 10 or with exponentially grown SP at a MOI of 50. After 40 min of infection, a combination of 1 mg/ml flucloxacillin and 25 μg/ml lysostaphin or penicillin (100 units/ml) and streptomycin (100 μg/ml), were added to kill all extracellular SA or SP, respectively. Infected cells were harvested 30 min and 3 h after addition of antibiotics, washed twice with PBS, lysed with ddH_2_O, serially diluted, and drop plated. Phagocytosis was analyzed and calculated relative to the inoculum and intracellular survival was analyzed and calculated relatively to the invasion (30min time point).

To analyze intracellular survival of the bacteria within monocytes, isolated monocytes were seeded into 24-well plates (TPP), pre-stimulated with 10% plasma (see above) and infected with exponentially grown SA or SP at a MOI of 50. After 40 min of infection, a combination of 1 mg/ml flucloxacillin and 25 μg/ml lysostaphin or penicillin (100 units/ml) and streptomycin (100 μg/ml), were added to kill all extracellular SA or SP, respectively. Infected cells were harvested 30 min and 90 min after addition of antibiotics, washed twice with PBS, lysed with 0.02% of Triton X-100 in ddH_2_O, serially diluted and drop plated. Phagocytosis was analyzed and calculated relative to the inoculum and intracellular survival was analyzed and calculated relatively to the invasion (30min time point). End-point supernatant from both neutrophil and monocyte bacterial infection experiments were collected, filter-sterilized (0.22 μ) and utilized for cytokine measurement.

### Flow cytometry

Staining of reactive oxygen species (ROS, for neutrophils and monocytes) and nitric oxide (NO, for monocytes) was performed after 1 h of bacterial challenge or plasma stimulation only by incubation with 5 μM CellROX green reagent and 5 μM DAF-FM diacetate (ThermoFisher) respectively, for 30 min. After the incubation period, cells were washed with DPBS. Cells were stained with either LIVE/DEAD fixable Near-IR or Aqua stain (ThermoFisher) in DPBS for 25 min at 4°C. Next, cells were washed with FACS buffer and stained for surface antigens for 30 min at 4°C. Antibodies included anti-CD15 eFluor450 (clone: HI98), anti-CD181 FITC (8F1-1-4), anti-CD182 PerCP-eFluor710 (5E8-C7-F10), anti-CD183 PE-eFluor610 (CEW33D), anti-CD66b APC (G10F5), anti-HLA-DR eFluor450 (LN3), anti-CD45 eFluor506 (HI30), anti-CD14 SB600 (61D3), anti-CD64 FITC (10.1), anti-CD163 PerCP-eFluor710 (GHI/61), anti-CD16 PE (CB16), anti-CD86 PE-Cyanine 5.5 (IT2.2), anti-CD206 PE-Cyanine 7 (19.2), anti-CD169 APC (7–239), anti-CD11b AF700 (VIM12), anti-CD3 APC-eFluor780 (UCHT1), anti-CD19 APC-eFluor780 (HIB19), anti-CD56 APC-eFluor780 (CMSSB), anti-CD119 FITC (BB1E2) and anti-CX_3_CR1 APC (2A9-1) from ThermoFisher, anti-CD195 BV510 (J418F1), anti-CD184 BV605 (12G5), anti-CD191 PE-Cyanine7 (5F10B29), anti-CD88 AF700 (S5/1), anti-CD192 PerCP-Cyanine5.5 (K036C2), anti-CD80 PE-Dazzle594 (2D10) and anti-CD120b PE-Cyanine7 (3G7A02) from Biolegend. For intracellular MPO staining, neutrophils were washed, fixed and permeabilized with the Cytofix/Cytoperm Fixation/ Permeabilization Solution Kit (BD) for 15 min at 4°C and stained subsequently for another 30 min with anti-MPO eFluor450 (455-BE6). To assess neutrophil extracellular traps (NETs), cells were stained first with LIVE/DEAD fixable Aqua, followed by staining for surface antigens as described above, after which they were washed with DPBS and subsequently stained with SYTOX Green in DPBS for 30min. To stain for extracellular MPO-DNA complexes, neutrophils were stained exactly as described for NETs with the addition of the MPO staining during the surface antigen step. Cells were analyzed on an Attune NxT (ThermoFisher). All antibodies and concentrations used are listed in S3 Table. Flow cytometry data were analyzed with FlowJo (v10.2). Neutrophils and monocytes were gated based on their forward- and side-scatter properties, single cells and ultimately live cells. Neutrophils were characterized as CD66b+CD16+, or CD66b+CD15+, whereas monocytes were divided into subgroup based on CD14+CD16- (classical), CD14+CD16+ (intermediate) and CD14dimCD16+ (non-classical) for further analysis.

### Microscopy and NETs quantification

Neutrophils were stimulated as described above and placed within wells of a μ-slide (iBidi) and centrifuged at 200g for 2 min, after which they were challenged with *S*. *aureus* for 1.5 h. NETs were stained by directly adding SYTOX Green and 2μM Hoechst 33342 (ThermoFisher) for 30min at room temperature to the wells. The confocal laser scanning microscopy images were obtained with a Leica TCS SP8 inverted microscope using a 63×/1.4NA oil immersion objective. The whole wells were inspected for NETs formation and two to three representative spots per condition were imaged. The obtained images were processed using Imaris 9.2.0 software (Bitplane) to obtain tifs for further analysis. Other standard light microscopy images of fixed cells were obtained on a fully automated Olympus IX83 with a 40X objective (UPLFLN40XPH-2) illuminated with a PE-4000 LED system through a quadband filter set (U-IFCBL50). Sixteen positions per sample were assigned before the sample was prepared to avoid potential experimenter bias. Automated NET quantification was performed as described in S4 Fig: after filtering nuclei on DAPI signal (threshold set manually for each 8-samples experiment), extracellular DNA was quantified on Sytox Green signal. Images containing large cell aggregates that could not be resolved were discarded. Nuclei were counted after watershed segmentation on the DAPI mask. Images were processed using ImageJ software (Rasband, W.S., ImageJ, U. S. National Institutes of Health, Bethesda, Maryland, USA, https://imagej.nih.gov/ij/, 1997–2018) and Matlab R2020a (MathWorks).

### Plasma protein determination by LC-MS

Protein levels in patient and healthy donors’ plasma were assessed by a proteomics approach using an Evosep One (Evosep) coupled to a TIMS TOF Pro (Bruker). The proteins were enriched from plasma by using the Agilent StrataClean resin (Agilent) and a modified version of the protocol used by Nagel et al., 2018 [93]. In brief, 20 μl/sample of StrataClean resin were aliquoted in 1.5ml low protein binding tubes (Sarsted), washed with ET buffer (50 mM Tris, 10 mM EDTA, pH 7) and primed by digestion with 12 M HCL at 100°C for 16 h. After priming, the beads were washed twice with TE buffer and added directly to aliquoted plasma in 1.5 ml low protein binding tubes. Every sample treated with the primed and washed Strataclean resin was incubated overnight at 4°C using an overhead rotator. After the initial incubation, the samples were centrifuged at 8000 g for 20 min, the plasma was transferred to a new low protein binding tube while the beads were washed twice with ET buffer and vacuum dried. A second treatment with Strataclean resin was performed on the plasma recovered from the first round. Next, the plasma supernatant was discarded and the resin fraction was washed twice with ET buffer, pooled together with the first fraction of beads and vacuum dried. Dried samples were sent to the Functional Genomic Center Zurich for digestion, injection and analysis.

For each sample, the washed StrataClean resin were re-suspended in 45 μl digestion buffer (triethylammonium bicarbonate (TEAB), pH 8.2), reduced with 5 mM TCEP (tris(2-carboxyethyl)phosphine) and alkylated with 15mM chloroacetamide. Proteins were on-bead digested using 5μl of Sequencing Grade Trypsin (100 ng/μl in 10 mM HCl, Promega). The digestion was carried out at 37°C overnight. The supernatants were transferred to new tubes and the beads were washed with 150 μl trifluoroacetic acid (TFA) -buffer (0.1% TFA, 50% acetonitrile). These supernatants were collected and combined with the previously collected one. The samples were dried to completeness and re-solubilized with 20 μl of 3% acetonitrile, 0.1% formic acid for MS analysis. For MS analysis, 200 μg peptides were loaded on an Evotips following the provided instructions (Evosep).

MS analyses were performed on an Evosep One (Evosep) coupled to the TIMS TOF Pro (Bruker). For LC-MS/MS injections, samples were separated with the Evosep method “30 samples/day” keeping the analytical column [PSC-15-100-3-UHPnC, ReproSil c18 3 μm 120A 15 cm ID 100 μm, PepSep) at 50°C. For the dual TIMS TOF, MS were scanned from m/z 100 to m/z 1700 in ddaPASEF mode (data dependent acquisition Parallel Accumulation Serial Fragmentation [94]. For the ion mobility settings, the inversed mobilities from 1/K0 0.60 Vs/cm^2^ to 1.60 Vs/cm^2^ were analyzed with ion accumulation and ramp time of 166 ms, respectively. One survey TMS-MS scan was followed by ten PASEF ramps for MS/MS acquisition, resulting in a 1.9 sec cycle time. Singly charged ions were excluded using the polygon filter mask and isolation windows for MS/MS were set to m/z 2.0 for precursor ions below *m/z* 700, and m/z 3.0 for ions above. The mass spectrometry proteomics raw data have been deposited to the ProteomeXchange Consortium via the PRIDE [95] partner repository with the dataset identifier PXD029547.

Protein quantification was performed using label free quantification and match between run option with FragPipe (version 16.0), MSFragger (version 3.3) and Philosopher (version 4.4.0) [96]. Spectra were searched against the reviewed Uniprot Human (UP000005640) merged with the SarsCovid (UP000464024) database (downloaded 20210825), containing common contaminants and decoys. For the closed search settings, strict Trypsin digestion with max. two missed cleavages was defined. Carbamidomethylation of cysteine was set as fixed modification, while methionine oxidation and N-terminal protein acetylation were set as variable.

The statistical analysis was implemented in R [97] using the package prolfqua [98].

For further downstream analyses, we use the protein intensity estimates reported in the combined_protein.tsv output. We only report proteins with at least two identified peptides. For analysis, intensities stored in the columns with the suffix "Intensity" (razor intensities) were used. The protein intensities were log2 transformed, and we applied sample-wise z transformation to remove systematic abundance differences among samples, due to different sample loads on the LC column. After scaling, all have had a similar intensity distribution. We fitted a linear model to test for differences among conditions. Explanatory variables used are the factor condition with levels recovery and acute, factor wave with levels w1 (wave 1) and w2 (wave 2), and the interaction between these two factors.

We fitted the same linear model using the R lm function to all proteins. Then, we determined the contrasts (patient in acute- vs rec-phase, wave 1 vs. wave 2) between conditions based on the parameter estimates of the linear model. Afterward, the empirical Bayes variance shrinkage method implemented in the package limma was applied to moderate the t-statistics [99]. Finally, using the Benjamini-Hochberg method, we inferred the false discovery rate (FDR) from the p-values. Gene set enrichment analysis (GSEA) was done to determine up- and down- regulated pathways across conditions. For that, we ranked the proteins based on the previously computed t-statistics. We used the Reactome database to determine the pathways [100–105]. GSEA was performed in R using the package fgsea [106], and multiple-testing correction on the pathways was also achieved by means of the Benjamini-Hochberg method.

Protein samples processing and final data analysis was performed at the Functional Genomic Center Zurich (FGCZ), University of Zurich/ETH Zurich.

### Statistical analysis

The number of donors is annotated in the corresponding figure legend. Differences between two groups were evaluated using either Mann-Whitney test or Wilcoxon signed-rank test. Kruskal-Wallis test with Dunn’s multiple comparisons test was used to evaluate differences among the three groups in all the analyses (GraphPad version 8.1.1). Differences between two groups as represented by contingency graph were evaluated using Chi-square test. Pearson test was used for correlations of normally distributed binary data. Significance level with p<0.05 are depicted in individual graphs.

For the statistical analyses involving several cytokines, measured cytokine values were normalized based on the standard z-score formula. This allowed to compare cytokines to each other and to obtain a sum of z-scores per patient.

## Supporting information

S1 Table Clinical characteristics of the patients. (PDF)

S2 Table Treatment received by the patients in COVID-19 wave 1. (PDF)

S3 Table List of antibodies and concentrations used in this study. (PDF)

S4 Table Proteomics table wave 1. (XLSX)

S5 Table Proteomics table wave 2. (XLSX)

S6 Table Proteomics table comparison wave 1 vs wave 2. (XLSX)

S1 Fig Cytokine levels in plasma from COVID-19 patients in acute- (n = 25) or rec-phase (n = 19) as well as healthy donors (n = 17). Twenty-four different cytokines, grouped as neutrophil effectors, monocyte effectors and pro-inflammatory and anti-inflammatory mediators, were determined from plasma using a luminex multiplex assay. Data presented as whisker plots with box indicating interquartile range and error bars indicating highest and lowest value. p values were determined by using Mann-Whitney test.
(TIF)

S2 Fig (**A**) PCA of healthy donors (white, n = 17), acute- (red, n = 21) and rec-phase (blue, n = 18) COVID-19 patients grouping the plasma cytokine levels and status of secondary bacterial infections (superinfection). In order to control for a potential bias (i.e., presence of any bacterial superinfection at the time of sampling-four patients), the PCA and normalized cytokine values (sum of Z-scores) values were replotted wherein we removed those four patients secondarily. Patients who developed superinfections are depicted as triangle and patients without superinfection as circle symbols. (**B**) PCA of healthy donors (white, n = 17) and acute-phase (red, n = 21). Patients who developed superinfection are depicted as triangle and patients without superinfection as circle symbols (**C**) PCA of healthy donors (white, n = 17), and rec-phase (blue, n = 18). Patients with superinfection are depicted as triangle and patients without superinfection as circle symbols (**D**) Normalized cytokine values (sum of Z-scores) in the plasma of acute- (red) or rec-phase (blue) patients with or without bacterial superinfection and healthy donors (white). Data presented as box plots with box indicating interquartile range and error bars indicating highest and lower value. p values were determined by using Mann-Whitney test. (**E**) Integrated correlation clustering map of relevant clinical parameters; circle-color indicates positive (blue) and negative (red) correlations, color intensity represents correlation strength as measured by the Pearson’s correlation coefficient, with asterisks delineating statistical significance. CCI; Charlson Comorbidity Index, USZ; University Hospital Zurich.
(TIF)

S3 Fig Phagocytosis capacity of COVID-19 patient or healthy donor neutrophils (left) and monocytes (right) pre-exposed to 10% COVID-19 plasma (solid symbols) or healthy donor plasma for 3h (open symbols) and subsequently challenged with SP (**A and B**) or SA at MOI 10 (**C and D**). **(E-H)** Intracellular killing capacity of COVID-19 patient (acute-phase: red and rec-phase: blue) or healthy donor neutrophils **(E and G)** and monocytes **(F and H)** (n = 8–10) pre-exposed to 10% patient plasma for 3 h and subsequently infected with SP **(E and F)** or SA **(G and H)** at MOI 10. **(I and J)** Phagocytosis rate (**I**) and intracellular bacterial survival (**J**) of healthy donor monocytes pre-exposed to 10% COVID-19 plasma (solid symbols) or healthy donor plasma (open symbols) for overnight and subsequently challenged with SA or SP at MOI 10. Each symbol represents cells from one human subject, stimulated with either COVID-19 or healthy donor plasma from one donor. For panels **E** and **F**, data are presented as the mean value ± SEM. For panels (**A-D**) p values were determined by using Wilcoxon signed-rank test without adjustment for multiple testing. For panels (**E-J**) p values were determined by using Mann-Whitney test.
(TIF)

S4 Fig Functional characterization of COVID-19 patient’s (acute-phase: red and rec-phase: blue) or healthy donor’s neutrophils pre-exposed to 10% COVID-19 plasma (solid symbols) or healthy plasma (open symbols) for 3 h and subsequently challenged with either SA or SP at MOI 1 for 1 h. (**A and B**). Representative histogram of quantification of intracellular MPO in acute-phase patients (**A** (SA) and **B** (SPN)), (**C**) Cell viability as determined by AQUA positive cells, COVID-19 patients (acute-phase) or healthy donor’s neutrophils pre-exposed to 10% COVID-19 plasma (solid symbols) or healthy plasma (open symbols) (n = 7–9) and compared to healthy donors. Each symbol represents cells from one human subject, exposed to either COVID-19 or healthy donor plasma from one donor. (**D**) Representative flow cytometry plots of cell viability as determined by AQUA positive cells. For panel C, p values were determined by using Mann-Whitney test.
(TIF)

S5 Fig **(Aa-Ac)** Image analysis process used to quantify NETs from microscopy images (a) using Hoechst (b) and SYTOX green images (c). (**Ad)** First, flat-field correction is applied to Hoechst images in order to get rid of vignetting. (**Ae**) A mask is then obtained through thresholding and dilating the binary image obtained. (**Af**). Nuclei are then quantified after filtration of the small objects resulting from auto-fluorescence of plasma particles and *S*. *aureus* cells and watersheding (**Ag**). The NETs mask is obtained after applying the Hoechst mask to the SYTOX Green images, and then thresholding and filtering out small objects (**Ah**). **B)** Representative images of quantified NETs. Each COVID 19 patient / healthy donor (H.D.) pair is shown on a line with the four possible plasma / cells permutations. Each montage of three pictures is generated from the most representative image in terms of NETs area per Nuclei values, out of the 16 images obtained per sample, at randomized positions. The montage consists of the Hoechst and Sytox green overlay (**left**) and the resulting nuclei mask (**top right**) and the obtained NETs mask (**bottom right**).
(TIF)

S6 Fig (**A**) Characterization of COVID-19 monocyte subtypes distribution based on their cell surface expression of CD14 and CD16. Data are presented as the mean value ± SEM. (**B-F**) Representative histogram of ROS production (**B**) and normalized ROS (**C and D**) and NO GMFI values (**E and F**) by COVID-19 or healthy donor monocytes pre-exposed to 10% COVID-19 plasma (solid symbols) or healthy donor plasma (open symbols) for 3 h and subsequently challenged with SA (**C and E**) or SP (**D and F**) at MOI 1 (**G and H**) ROS production by non-classical monocytes pre-exposed and challenged with SA (**G**) or SP (**H**) as described in **B-F**. Each symbol represents cells from one human subject, stimulated with either COVID-19 or healthy donor plasma from one donor. For panel **A**, p values p values were determined by using Mann-Whitney test and for panels **C-H**, p values were determined by using Wilcoxon signed-rank test.
(TIF)

S7 Fig (**A and B**) Gating strategy for the quantification of NETs production in neutrophils by Flow-cytometry using the cell markers: CD66b^+^, CD15^+^, Live-dead fixable Aqua^-^ and SYTOX green^+^—MPO^+^. (**C-F**) Characterization and quantification of surface receptors CXCR3 (**C**), CXCR4 (**D**), CCR3 (**E**) and C5aR (**F**) in COVID-19 or healthy donor neutrophils pre-exposed to 10% of COVID-19 plasma (solid symbols) or healthy donor plasma (open symbols) for 3 h and subsequently challenged with SA or SP at MOI 1 or left unchallenged. Each symbol represents cells from one human subject, stimulated with either COVID-19 or healthy donor plasma from one donor. For panels C-F, p values were determined by using Mann-Whitney test.
(TIF)

S8 Fig **(A and B)** Flow cytometric determination of the expression of key surface markers in CD14^+^ CD16^-^ classical (**A**) and CD14^dim^ CD16^+^ non-classical monocytes (**B**) from COVID-19 acute- (red) or rec-phase (blue) as well as healthy donors (white), pre-exposed to 10% COVID-19 plasma (solid symbols) or healthy donor plasma (open symbols) for 3 h and subsequently challenged with SA or SP at MOI 1 or left unchallenged. (**C**) PCA of cell surface phenotype of COVID-19 patients acute- (red) or rec-phase (blue) and healthy donors’ (white) non-classical monocytes at the basal level without bacterial challenge, upon SA infection or SP infection. Each symbol represents cells from a single COVID-19 patient or healthy donor exposed to either COVID-19 or healthy donor plasma from one donor. For panels **A** and **B**, p values were determined by using Mann-Whitney test.
(TIF)

S9 Fig **(A and B)** Normalized cytokine values (sum of Z-scores) in the plasma of acute- (red) and rec-phase (blue) patients with (triangle) or without (circle) bacterial superinfection from first wave **left**) and second wave (**right**). Data presented as whisker plots with box indicating interquartile range and error bars indicating highest and lower value. For panels A-B, p values were determined by using Mann-Whitney test.
(TIF)

S10 Fig ROS production (**A**) and cell death (**B**) of healthy donor neutrophils (n = 5–7) pre-exposed to the indicated single (**A, B, C**) or combination of cytokines for 4 h (**D**) and subsequently infected with SA at MOI 1 (A and B) for 2 h. (**C** and **D**) Cell death of healthy donor neutrophils (n = 5–7) only exposed to the indicated single (**A**) or combination of cytokines for 4 h (**D**). Cocktail 1, TNF-α and IFN-γ; cocktail 2, IL-6, IL-8, G-CSF and IFN-α; cocktail 3, IL-4, IL-10 and IFN-γ; cocktail 4, IL-4, IL-6, IL-8, IL-10, TNF-α, IFN-α, IFN-γ, G-CSF, SDF-1α, MIP-1α and MIP-1β. Each symbol represents neutrophils from one healthy donor. Data are presented as the mean value ± SEM. (**E**) ROS production of healthy donor neutrophils (n = 5–8) pre-treated with receptor blockers or isotype controls and exposed to 10% acute-phase COVID-19 plasma (“Acute-phase plasma”, n = 6 plasmas). Employment of receptor blockers or isotype controls, pre-exposed to TNF-α and IFN-γ (“Cytokines”) with or without receptor blockers and 10% acute-phase COVID-19 or 10% healthy donor plasma (“Plasma”, n = 6 plasmas) for 4 h and subsequently infected with SA at MOI 1. Each symbol represents cells from one healthy donor. For graphs presented in B-D, p values were determined by using Mann-Whitney test, for E, by using Kruskal-Wallis test with Dunn’s multiple comparison test and Mann-Whitney test.
(TIF)

S11 Fig Intracellular bacterial survival in healthy donor monocytes (n = 9) pre-exposed to the indicate single (**A**) or combination of cytokines for 18 h (**B**) and subsequently infected with SA at MOI 10 (A and B) or 1 (C). Cocktail 1, TNF-α and IFN-γ; cocktail 2, IL-6, IL-8, G-CSF and IFN-α; cocktail 3, IL-4, IL-10, IFN-γ and CX_3_CL_1_; cocktail 4, IL-4, IL-6, IL-8, IL-10, TNF-α, IFN-α, IFN-γ, G-CSF, SDF-1α, MIP-1α, MIP-1β and CX_3_CL_1_. For panels A and B p values were determined by using Mann-Whitney test.
(TIF)

S12 Fig (**A**) PCA analysis of the proteomic profiles from nine acute patients from wave 1 and eight acute patients from wave 2 and (**B**) changes in the representation of plasma proteins and their impact on GSEA analysis of metabolic and cellular immune defense pathways between acute- and rec-phase wave 1 vs acute- and rec-phase wave 2. A p-value of 0.05 and a minimum change of two folds and an FDR of 0.05 and 0.25 was considered for statistical significance. (**C**) heat map of proteins presenting significant changes in their intensity and classified by the patient status (acute- or rec-phase) and the time of sampling (wave 1 or wave 2). A p-value of 0.05 was considered for statistical significance and no FDR was used for the heat map determination.
(TIF)

S13 Fig (**A**) Phagocytosis capacity of healthy donor neutrophils (n = 6) pre-exposed to 10% of wave 1 (n = 12), wave 2 (n = 11) or healthy donor plasma (n = 9) for 4 h and subsequently challenged with SA at MOI 10. (**B**) Cell death of healthy donor neutrophils (n = 5) pre-exposed to 10% of wave 1 (n = 9), wave 2 (n = 11) or healthy donor plasma (n = 8) for 4 h. (**C-F**) Intracellular bacterial survival (**C**), ROS production (**D**), cell death (**E**) and HLA-DR expression (**F**) of healthy donor monocytes (n = 7) pre-stimulated with 10% of wave 1 (n = 12–14), wave 2 (n = 12–14) or healthy donor plasma (n = 11–12) and subsequently infected with SA. (**G and H**) HLA-DR expression (**G**) and cell death (**H**) of healthy donor monocytes (n = 7) pre-stimulated with 10% of wave 1 (n = 12), wave 2 (n = 12) or healthy donor plasma (n = 11) uninfected cells.
(TIF)

S14 Fig (**A**) Normalized cytokine values (sum of Z-scores) depicted as cytokine summary score (inflammatory index) in the plasma of rec-phase COVID-19 patients between first and second wave with or without superinfection. The cytokine summary score from first wave is also presented in Fig 1D. (**B-I**) Clinical as well as laboratory parameters that are part of the routine diagnostics including neutrophils (**B**), monocytes (**C**) and leukocytes counts (**D**), SAPS (**E**) and SOFA score (**F**), age (**G**), time to superinfection (**H**), LDH (**I**) and D-dimer levels (**J**) in acute-phase COVID-19 patients during first (n = 25) vs second wave (n = 38), with or without superinfection. Data are presented as the mean value ± SEM. SAPS, Simplified acute physiology score; SOFA, Sepsis-related organ failure assessment score; LDH, lactate dehydrogenase. P values determined using Mann-Whitney test.
(TIF)

S15 Fig (**A and B**). Percentage of identified species in bacterial superinfection cases among critically ill COVID-19 patients during wave 1 (**A**) and wave 2 (**B**). (**C-E**) Plasma titration assay using COVID-19 acute-phase or healthy plasma at 1, 5, 10 or 20% in RPMI and neutrophils from healthy donors. Phagocytosis (**A**), intracellular survival (**B**) and ROS production (**C**) after infection with *Staphylococcus aureus* were determined from the different stimulations with plasma.
(TIF)
