# Supplementary material for: Hyperinflammatory environment drives dysfunctional myeloid cell effector response to bacterial challenge in COVID-19
Source: PLoS Pathog. 2022 Jan 10;18(1):e1010176. doi: 10.1371/journal.ppat.1010176 (PMC8782468; doi:10.1371/journal.ppat.1010176)
Supplement: S1 Table — (PDF) [file ppat.1010176.s001.pdf]

S1 Table: Clinical characteristics of the patients

Clinical Characteristics	1st Wave (March 2020 – June 2020)	2nd Wave (July 2020 – December 2020)
Study Patients	Study Patients (n=25)	Study Patients (n=38)
Age (mean ± SD)	60.3 (±9.0)	63.2 (±13.4)
Male (%)	20 (80%)	27 (71%)
Diabetes (%)	12 (48%)	12 (32%)
COPD/Asthma (%)	5 (20%)	4 (11%)
Charlson Comorbidity Index (median)	1.0	1.0
Malignancy (%)	3 (12%)	3 (8%)
Immunosuppression (%)	4 (16%)	2 (5%)
28 Day Survival (%)	22 (88%)	31 (82%)
Alive at ICU Discharge (%)	20 (80%)	31 (82%)
Bacterial Superinfection (%)	16 (64%)	21 (55%)
Mechanical Ventilation (%)	24 (96%)	36 (95%)
Length of Mechanical Ventilation (mean ± SD)	22.2* (±21.6)	11.7 (±11.1)
Length of ICU-Stay (mean ± SD)	29.0* (±27.0)	14.6 (±12.6)
SOFA Score Day 1 (median)	8.0	7.0
SAPS on Admission (median)	36	36.0

COPD, Chronic Obstructive Pulmonary Disease; ICU, Intensive Care Unit; SD, Standard deviation; SOFA, Sepsis-related organ failure assessment score; SAPS, Simplified Acute Physiology Score.

* One patient received dexamethasone at a later time point after the experimental sampling and therefore was excluded from temporal analyses.
