# Supplementary material for: Hyperinflammatory environment drives dysfunctional myeloid cell effector response to bacterial challenge in COVID-19
Source: PLoS Pathog. 2022 Jan 10;18(1):e1010176. doi: 10.1371/journal.ppat.1010176 (PMC8782468; doi:10.1371/journal.ppat.1010176)
Supplement: S2 Table — (PDF) [file ppat.1010176.s002.pdf]

S2 Table: Treatment received by the patients in COVID-19 wave 1 and wave 2

| Wave 1 | Acute phase (n=25) | | | | Recovery phase (n=19) | | | |
|---|---|---|---|---|---|---|---|---|
| | Anti-infectives | Hydroxy-chloroquin | Steroids | Other Immuno-suppression | Anti-infectives | Hydroxy-chloroquin | Steroids | Other Immuno-suppression |
| Total | 24/25 (96%) | 11/25 (44%) | 7/25 (28%) | 4/25 (16%) | 12/19 (63%) | 0/20 (0%) | 7/19 (36%) | 3/19 (16%) |

| Wave 2 | Acute phase (n=38) | | | | Recovery phase (n=21) | | | |
|---|---|---|---|---|---|---|---|---|
| | Anti-infectives | Hydroxy-chloroquin | Steroids | Other Immuno-suppression | Anti-infectives | Hydroxy-chloroquin | Steroids | Other Immuno-suppression |
| Total | 37/38 (97%) | 0/38 (0%) | 38/38 (100%) | 3/38 (7.9%) | 9/21 (42%) | 0/21 (0%) | 10/21 (47.6%) | 1/21 (4.7%) |

Definitions:

Anti-infective: Use of either/or antibiotics, antivirals, antifungals in the last 48 hours before sampling. The use of prophylactic anti-infective treatment (e.g. trimethoprim-sulfamethoxazole 3x/week) was not considered in this analysis.

Hydroxychloroquin: Use of hydroxychloroquin in the last 7 days before sampling

Steroids: Any use of steroids equivalent to 20mg prednisone per day in the last 48 hours before sampling
