# Supplementary material for: Hyperinflammatory environment drives dysfunctional myeloid cell effector response to bacterial challenge in COVID-19
Source: PLoS Pathog. 2022 Jan 10;18(1):e1010176. doi: 10.1371/journal.ppat.1010176 (PMC8782468; doi:10.1371/journal.ppat.1010176)
Supplement: S3 Table — (PDF) [file ppat.1010176.s003.pdf]

S3 Table: List of antibodies and concentrations used in this study

Neutrophils Intracellular effectors

Laser	Channel	Emission	Fluorochrome	Antigen	Clone	Dilution (1:x)	Manufacturer
Violet	450/50	445	eFluor 450	MPO	455-BE6	100	ThermoFisher
	525/50	526		LIVE/DEAD Fixable Aqua		500	ThermoFisher
Blue	530/30	517	FITC	CellROX Green		500	ThermoFisher
Yellow	586/15	576	PE	CD15	HI98	50	ThermoFisher

Neutrophils NETs

Laser	Channel	Emission	Fluorochrome	Antigen	Clone	Dilution (1:x)	Manufacturer
Violet	450/50	445	eFluor 450	MPO	455-BE6	100	ThermoFisher
	525/50	526		LIVE/DEAD Fixable Aqua		500	ThermoFisher
Blue	530/30	517	FITC	SYTOX Green	BB1E2	100	ThermoFisher
Yellow	586/15	576	PE	CD15	HI98	50	ThermoFisher

Neutrophils Panel

Laser	Channel	Emission	Fluorochrome	Antigen	Clone	Dilution (1:x)	Manufacturer
Violet	450/50	445	eFluor 450	CD15	HI98	50	ThermoFisher
	525/50	526	BV 510	CD195 (CCR5)	J418F1	50	Biolegend
	610/20	601	BV 605	CD184 (CXCR4)	12G5	50	Biolegend
Blue	530/30	517	FITC	CD181 (CXCR1)	8F1-1-4	50	ThermoFisher
	670/30	708	PerCP-eFluor 710	CD182 (CXCR2)	5E8-C7-F10	50	ThermoFisher
Yellow	586/15	576	PE	CD193 (CCR3)	12-1939-42	50	ThermoFisher
	610/20	606	PE-eFluor 610	CD183 (CXCR3)	CEW33D	50	ThermoFisher
	780/60	780	PE-Cy7	CD191 (CCR1)	5F10B29	50	Biolegend
Red	670/30	660	APC	CD66b	G10F5	50	ThermoFisher
	720/30	785	AF 700	CD88 (C5aR)	S5/1	50	Biolegend
	780/60	785		LIVE/DEAD Fixable Near-IR		750	ThermoFisher

Monocytes Intracellular effectors

Laser	Channel	Emission	Fluorochrome	Antigen	Clone	Dilution (1:x)	Manufacturer
Violet	525/50	526		LIVE/DEAD Fixable Aqua		500	ThermoFisher
	610/20	601	SB 600	CD45	2D1	100	ThermoFisher
Blue	530/30	517	FITC	CellROX Green/DAF-FM Diacetate		500/100	ThermoFisher
	670/30	708	PerCp	CD14	61D3	50	ThermoFisher
Yellow	586/15	576	PE	CD16	eBioCB16 (CB16)	50	ThermoFisher
	780/60	780	Pe-Cyanine7	HLA-DR	LN3	50	ThermoFisher

Monocytes Panel 1

Laser	Channel	Emission	Fluorochrome	Antigen	Clone	Dilution (1:x)	Manufacturer
Violet	450/50	445	eFluor 450	HLA-DR	LN3	50	ThermoFisher
	525/50	526	eFluor 506	CD45	HI30	50	ThermoFisher
	610/20	601	SB 600	CD14	61D3	50	ThermoFisher
Blue	530/30	517	FITC	CD64	10.1	50	ThermoFisher
	670/30	708	PerCP-eFluor 710	CD163	eBioGHI/61 (GHI/61)	50	ThermoFisher
Yellow	586/15	576	PE	CD16	eBioCB16 (CB16)	50	ThermoFisher
	695/40	561	PE-Cy5	CD86	IT2.2	50	ThermoFisher
	780/60	780	PE-Cy7	CD206 (MMR)	19.2	50	ThermoFisher
Red	670/30	660	APC	CD169 (Siglec-1)	7-239	50	ThermoFisher
	720/30	785	AF 700	CD11b	VIM12	50	ThermoFisher
	780/60	785	APC-eFluor 780	CD3	UCHT1	50	ThermoFisher
	780/60	785	APC-eFluor 780	CD19	HIB19	50	ThermoFisher
	780/60	785	APC-eFluor 780	CD56	CMSSB	50	ThermoFisher
	780/60	785		LIVE/DEAD Fixable Near-IR		750	ThermoFisher

Monocytes Panel 2

Laser	Channel	Emission	Fluorochrome	Antigen	Clone	Dilution (1:x)	Manufacturer
Violet	450/50	445	eFluor 450	HLA-DR	LN3	50	ThermoFisher

	525/50	526	eFLuor 506	CD45	HI30	50	ThermoFisher
	610/20	601	Super Bright 600	CD14	61D3	50	ThermoFisher
Blue	530/30	517	FITC	CD119	BB1E2	50	ThermoFisher
	670/30	708	PerCP-Cy5.5	CD192 (CCR2)	K036C2	50	Biolegend
Yellow	586/15	576	PE	CD16	eBioCB16 (CB16)	50	ThermoFisher
	620/15	606	PE-Dazzle 594	CD80	2D10	50	Biolegend
	780/60	780	PE-Cyanine7	CD120b	3G7A02	50	Biolegend
Red	670/30	660	APC	CX3CR1	2A9-1	50	ThermoFisher
	720/30	785	AF 700	CD11b	VIM12	50	ThermoFisher
	780/60	785	APC-eFluor 780	CD3	UCHT1	50	ThermoFisher
	780/60	785	APC-eFluor 780	CD19	HIB19	50	ThermoFisher
	780/60	785	APC-eFluor 780	CD56	CMSSB	50	ThermoFisher
	780/60	785		LIVE/DEAD Fixable Near-IR		750	ThermoFisher
