# Supplementary figures and images for: Hyperinflammatory environment drives dysfunctional myeloid cell effector response to bacterial challenge in COVID-19
Source: PLoS Pathog. 2022 Jan 10;18(1):e1010176. doi: 10.1371/journal.ppat.1010176 (PMC8782468; doi:10.1371/journal.ppat.1010176)

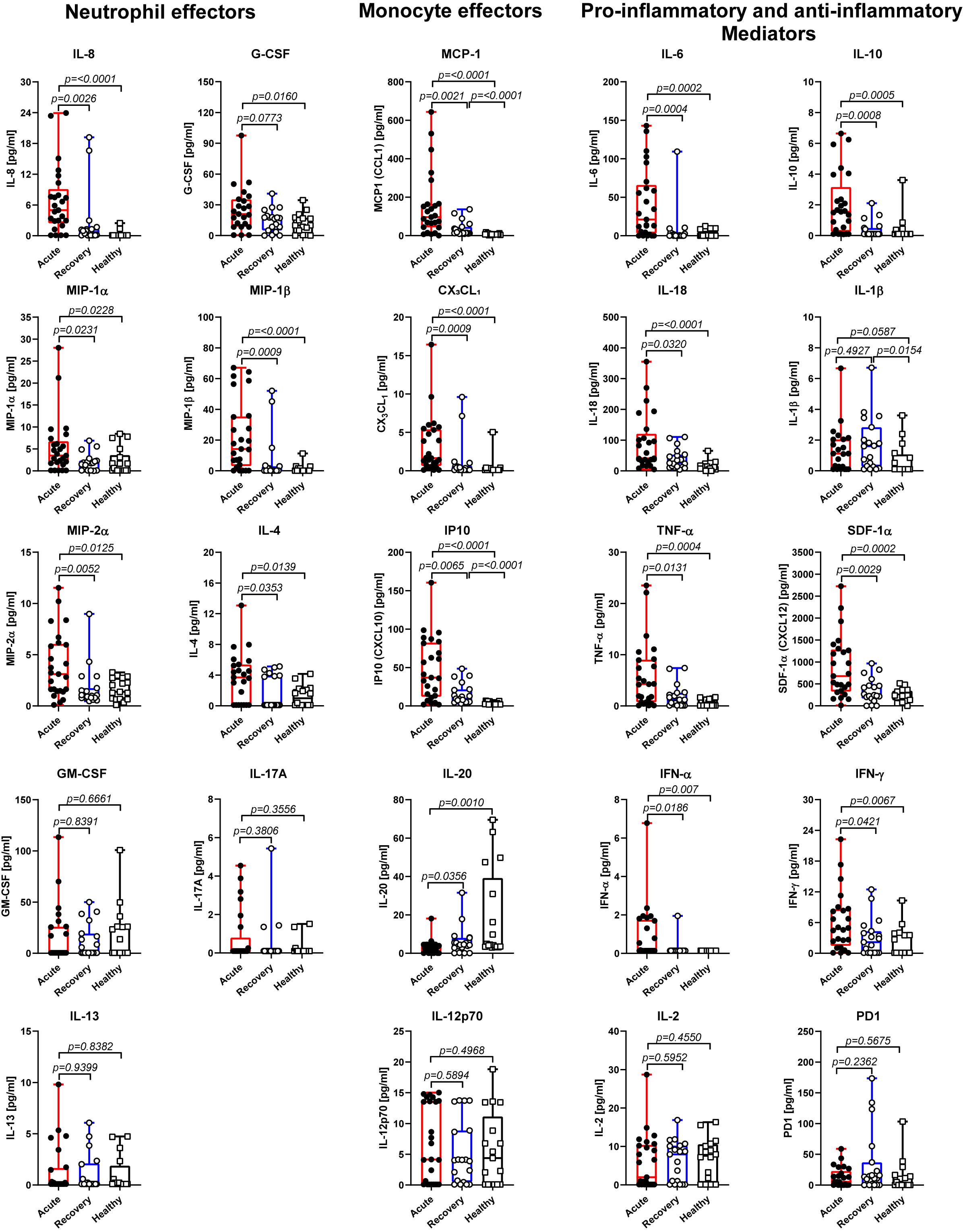

Supplement: S1 Fig — Cytokine levels in plasma from COVID-19 patients in acute- (n = 25) or rec-phase (n = 19) as well as healthy donors (n = 17). Twenty-four different cytokines, grouped as neutrophil effectors, monocyte effectors and pro-inflammatory and anti-inflammatory mediators, were determined from plasma using a luminex multiplex assay. Data presented as whisker plots with box indicating interquartile range and error bars indicating highest and lowest value. p values were determined by using Mann-Whitney test. (TIF) [file ppat.1010176.s007.tif]

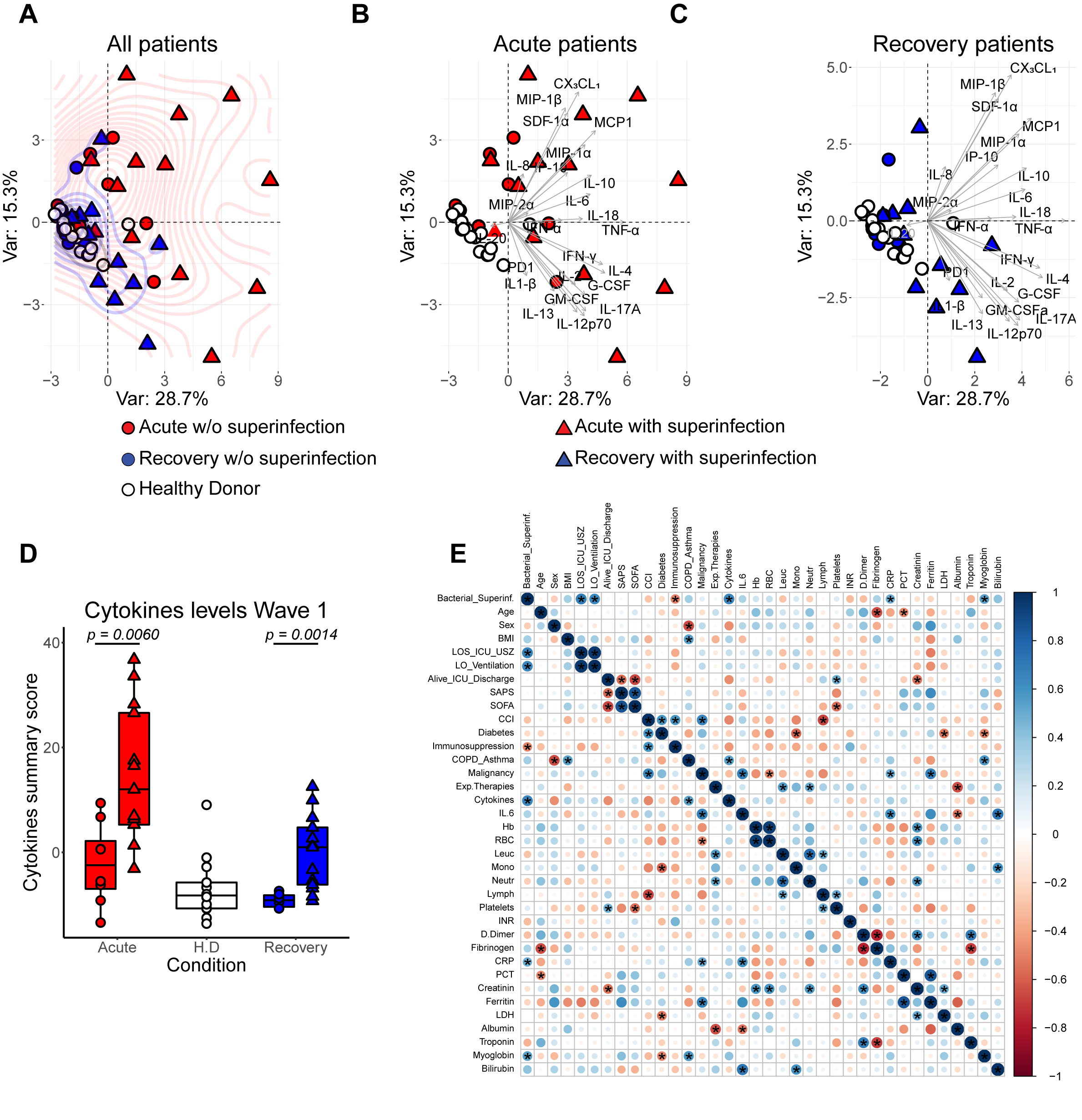

Supplement: S2 Fig — (A) PCA of healthy donors (white, n = 17), acute- (red, n = 21) and rec-phase (blue, n = 18) COVID-19 patients grouping the plasma cytokine levels and status of secondary bacterial infections (superinfection). In order to control for a potential bias (i.e., presence of any bacterial superinfection at the time of sampling-four patients), the PCA and normalized cytokine values (sum of Z-scores) values were replotted wherein we removed those four patients secondarily. Patients who developed superinfections are depicted as triangle and patients without superinfection as circle symbols. (B) PCA of healthy donors (white, n = 17) and acute-phase (red, n = 21). Patients who developed superinfection are depicted as triangle and patients without superinfection as circle symbols (C) PCA of healthy donors (white, n = 17), and rec-phase (blue, n = 18). Patients with superinfection are depicted as triangle and patients without superinfection as circle symbols (D) Normalized cytokine values (sum of Z-scores) in the plasma of acute- (red) or rec-phase (blue) patients with or without bacterial superinfection and healthy donors (white). Data presented as box plots with box indicating interquartile range and error bars indicating highest and lower value. p values were determined by using Mann-Whitney test. (E) Integrated correlation clustering map of relevant clinical parameters; circle-color indicates positive (blue) and negative (red) correlations, color intensity represents correlation strength as measured by the Pearson’s correlation coefficient, with asterisks delineating statistical significance. CCI; Charlson Comorbidity Index, USZ; University Hospital Zurich. (TIF) [file ppat.1010176.s008.tif]

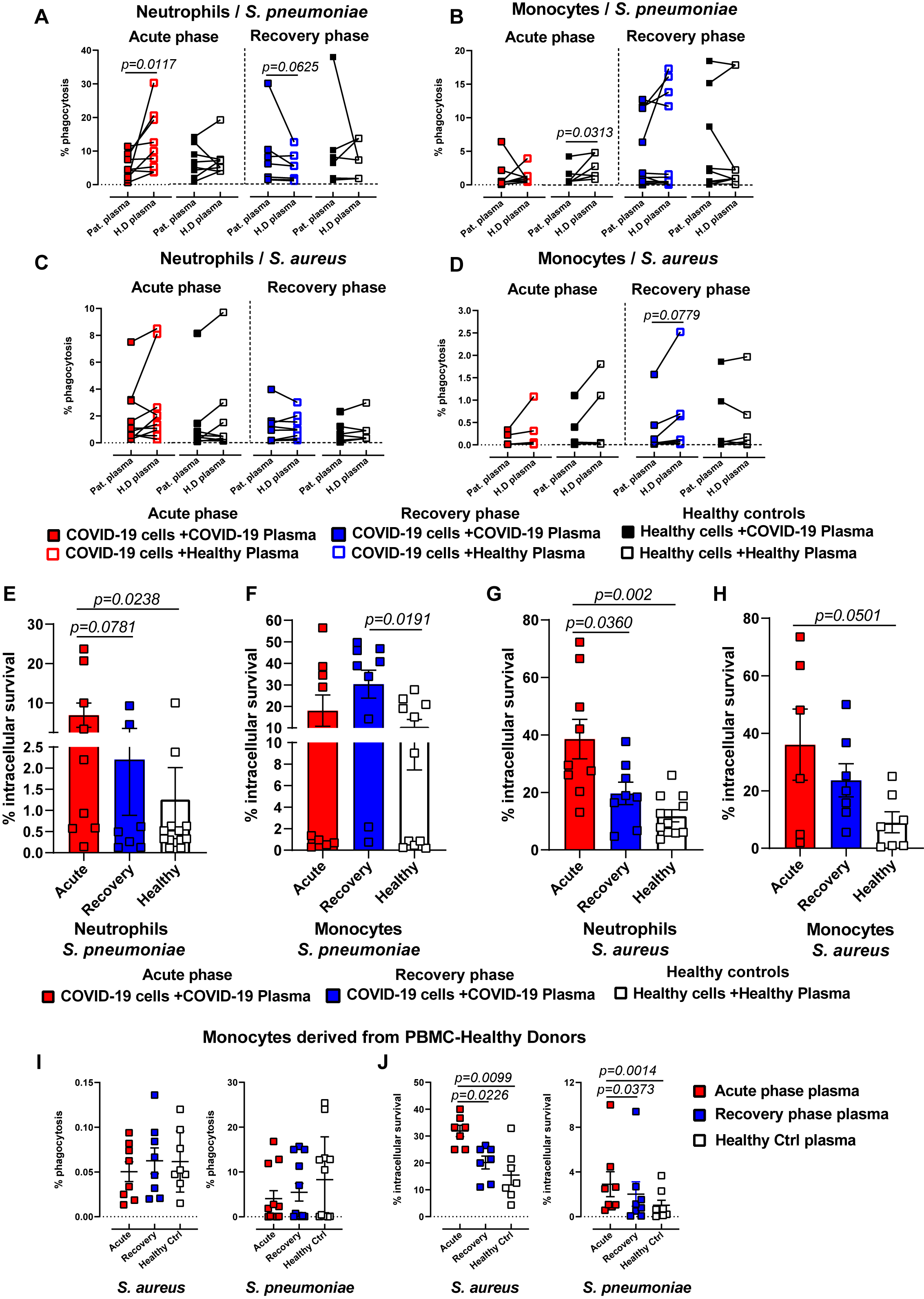

Supplement: S3 Fig — Phagocytosis capacity of COVID-19 patient or healthy donor neutrophils (left) and monocytes (right) pre-exposed to 10% COVID-19 plasma (solid symbols) or healthy donor plasma for 3h (open symbols) and subsequently challenged with SP (A and B) or SA at MOI 10 (C and D). (E-H) Intracellular killing capacity of COVID-19 patient (acute-phase: red and rec-phase: blue) or healthy donor neutrophils (E and G) and monocytes (F and H) (n = 8–10) pre-exposed to 10% patient plasma for 3 h and subsequently infected with SP (E and F) or SA (G and H) at MOI 10. (I and J) Phagocytosis rate (I) and intracellular bacterial survival (J) of healthy donor monocytes pre-exposed to 10% COVID-19 plasma (solid symbols) or healthy donor plasma (open symbols) for overnight and subsequently challenged with SA or SP at MOI 10. Each symbol represents cells from one human subject, stimulated with either COVID-19 or healthy donor plasma from one donor. For panels E and F, data are presented as the mean value ± SEM. For panels (A-D) p values were determined by using Wilcoxon signed-rank test without adjustment for multiple testing. For panels (E-J) p values were determined by using Mann-Whitney test. (TIF) [file ppat.1010176.s009.tif]

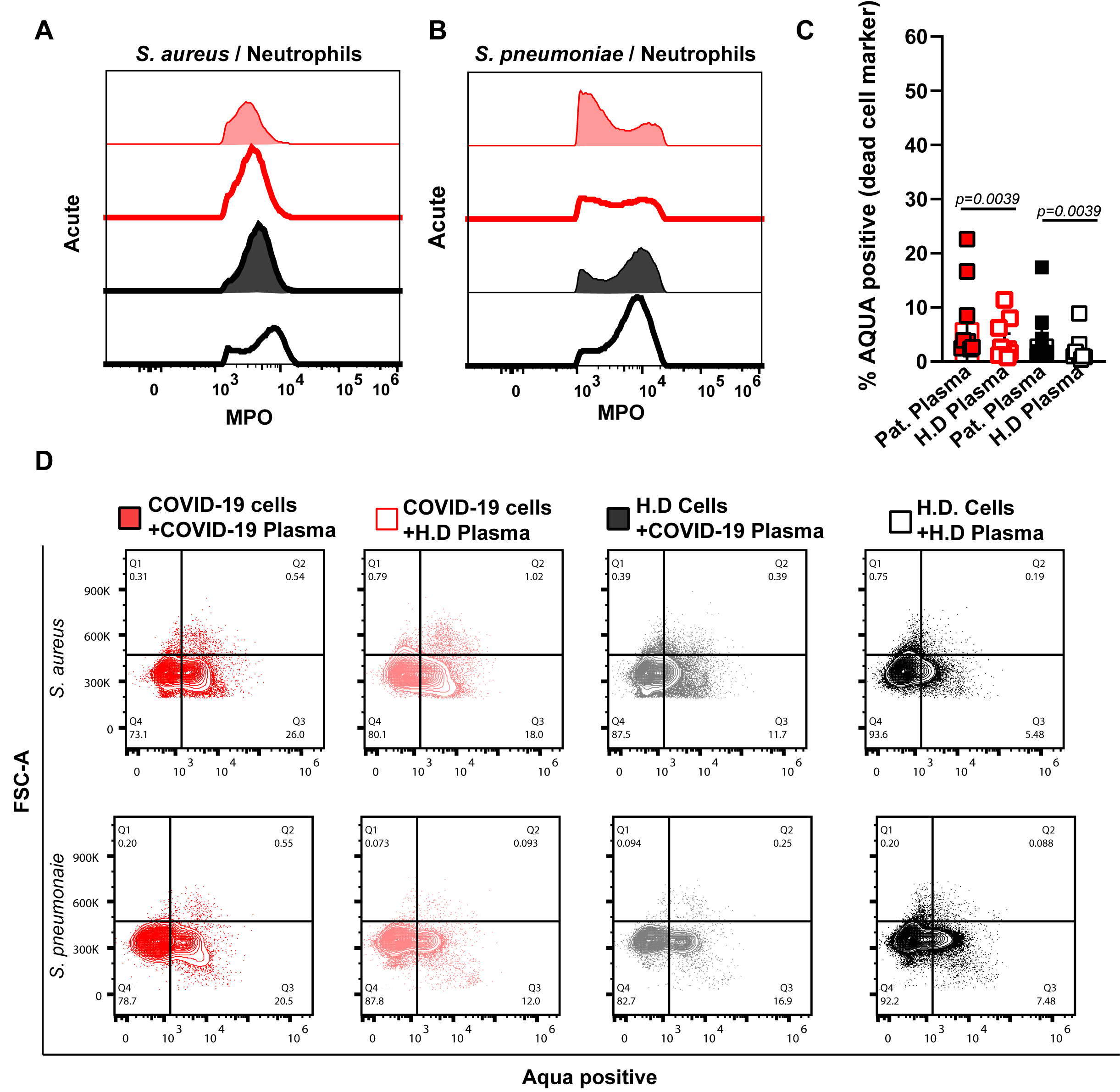

Supplement: S4 Fig — Functional characterization of COVID-19 patient’s (acute-phase: red and rec-phase: blue) or healthy donor’s neutrophils pre-exposed to 10% COVID-19 plasma (solid symbols) or healthy plasma (open symbols) for 3 h and subsequently challenged with either SA or SP at MOI 1 for 1 h. (A and B). Representative histogram of quantification of intracellular MPO in acute-phase patients (A (SA) and B (SPN)), (C) Cell viability as determined by AQUA positive cells, COVID-19 patients (acute-phase) or healthy donor’s neutrophils pre-exposed to 10% COVID-19 plasma (solid symbols) or healthy plasma (open symbols) (n = 7–9) and compared to healthy donors. Each symbol represents cells from one human subject, exposed to either COVID-19 or healthy donor plasma from one donor. (D) Representative flow cytometry plots of cell viability as determined by AQUA positive cells. For panel C, p values were determined by using Mann-Whitney test. (TIF) [file ppat.1010176.s010.tif]

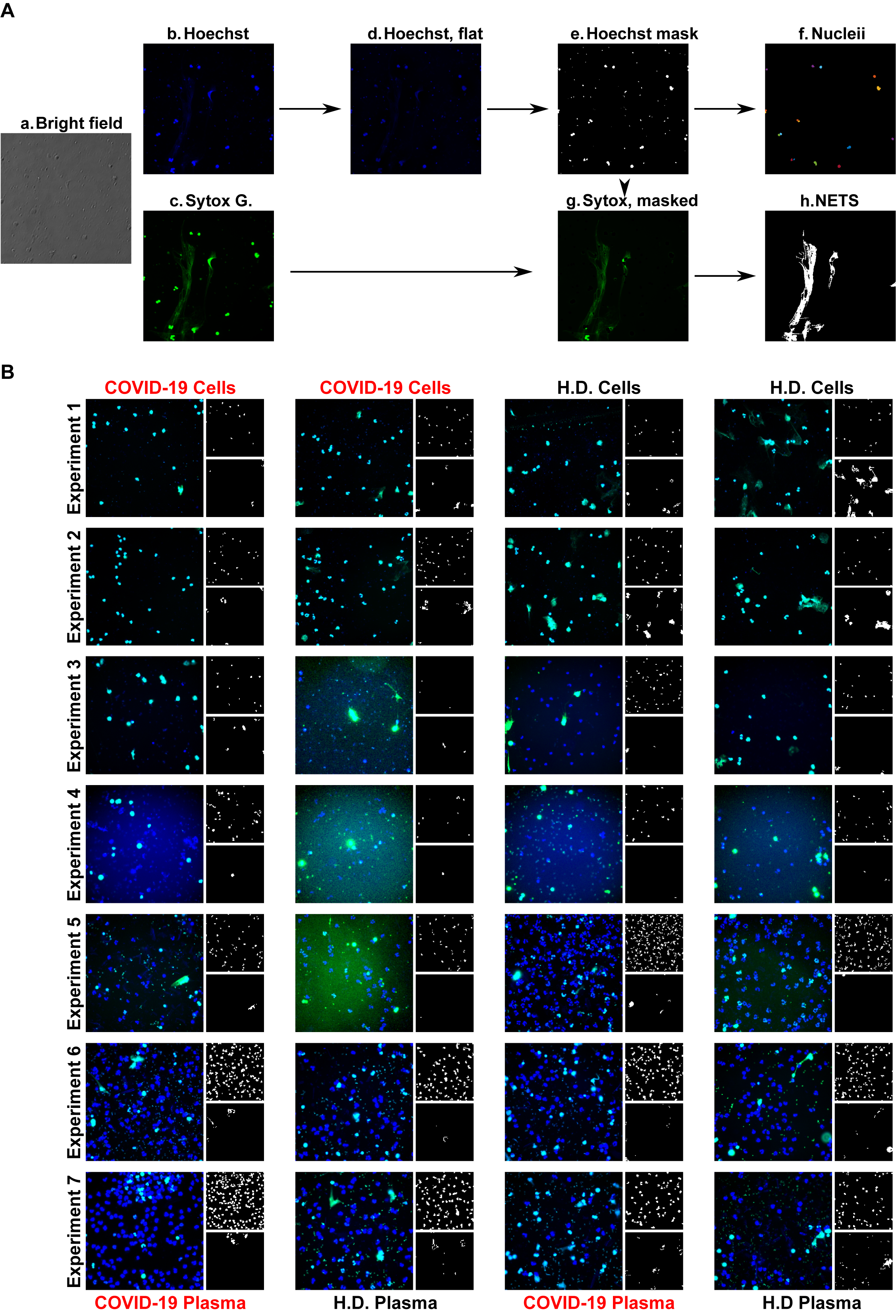

Supplement: S5 Fig — (Aa-Ac) Image analysis process used to quantify NETs from microscopy images (a) using Hoechst (b) and SYTOX green images (c). (Ad) First, flat-field correction is applied to Hoechst images in order to get rid of vignetting. (Ae) A mask is then obtained through thresholding and dilating the binary image obtained. (Af). Nuclei are then quantified after filtration of the small objects resulting from auto-fluorescence of plasma particles and S. aureus cells and watersheding (Ag). The NETs mask is obtained after applying the Hoechst mask to the SYTOX Green images, and then thresholding and filtering out small objects (Ah). B) Representative images of quantified NETs. Each COVID 19 patient / healthy donor (H.D.) pair is shown on a line with the four possible plasma / cells permutations. Each montage of three pictures is generated from the most representative image in terms of NETs area per Nuclei values, out of the 16 images obtained per sample, at randomized positions. The montage consists of the Hoechst and Sytox green overlay (left) and the resulting nuclei mask (top right) and the obtained NETs mask (bottom right). (TIF) [file ppat.1010176.s011.tif]

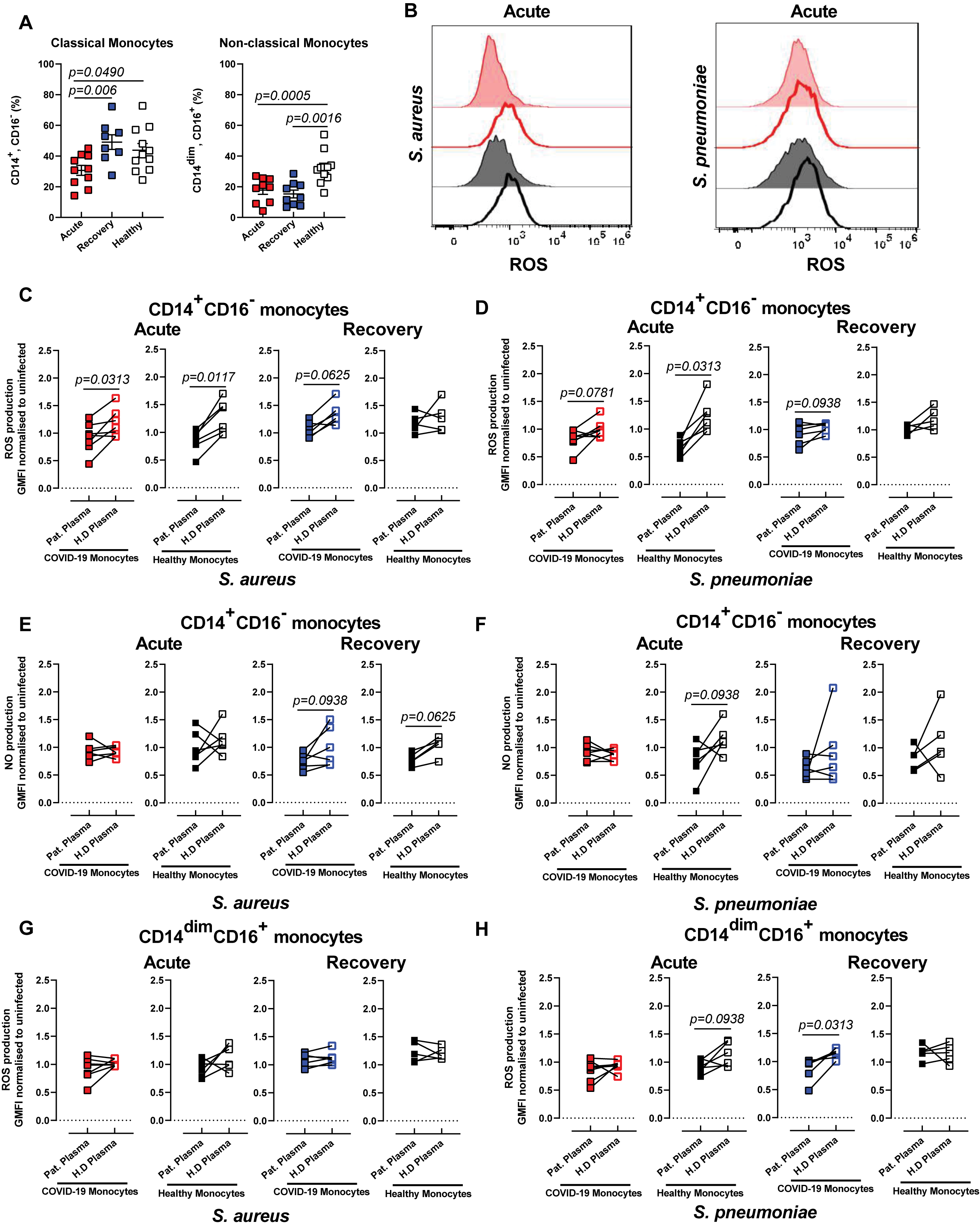

Supplement: S6 Fig — (A) Characterization of COVID-19 monocyte subtypes distribution based on their cell surface expression of CD14 and CD16. Data are presented as the mean value ± SEM. (B-F) Representative histogram of ROS production (B) and normalized ROS (C and D) and NO GMFI values (E and F) by COVID-19 or healthy donor monocytes pre-exposed to 10% COVID-19 plasma (solid symbols) or healthy donor plasma (open symbols) for 3 h and subsequently challenged with SA (C and E) or SP (D and F) at MOI 1 (G and H) ROS production by non-classical monocytes pre-exposed and challenged with SA (G) or SP (H) as described in B-F. Each symbol represents cells from one human subject, stimulated with either COVID-19 or healthy donor plasma from one donor. For panel A, p values p values were determined by using Mann-Whitney test and for panels C-H, p values were determined by using Wilcoxon signed-rank test. (TIF) [file ppat.1010176.s012.tif]

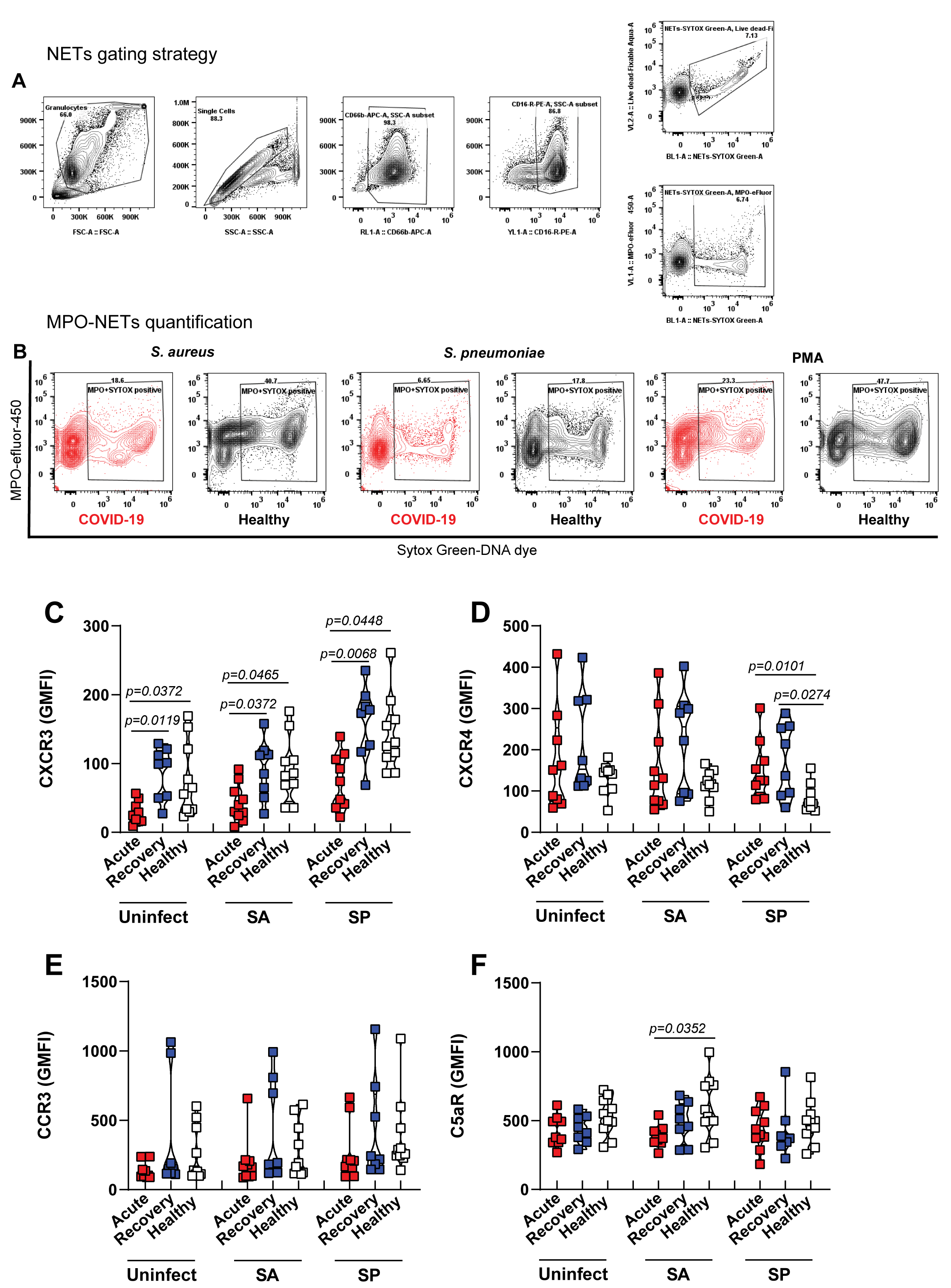

Supplement: S7 Fig — (A and B) Gating strategy for the quantification of NETs production in neutrophils by Flow-cytometry using the cell markers: CD66b+, CD15+, Live-dead fixable Aqua- and SYTOX green+—MPO+. (C-F) Characterization and quantification of surface receptors CXCR3 (C), CXCR4 (D), CCR3 (E) and C5aR (F) in COVID-19 or healthy donor neutrophils pre-exposed to 10% of COVID-19 plasma (solid symbols) or healthy donor plasma (open symbols) for 3 h and subsequently challenged with SA or SP at MOI 1 or left unchallenged. Each symbol represents cells from one human subject, stimulated with either COVID-19 or healthy donor plasma from one donor. For panels C-F, p values were determined by using Mann-Whitney test. (TIF) [file ppat.1010176.s013.tif]

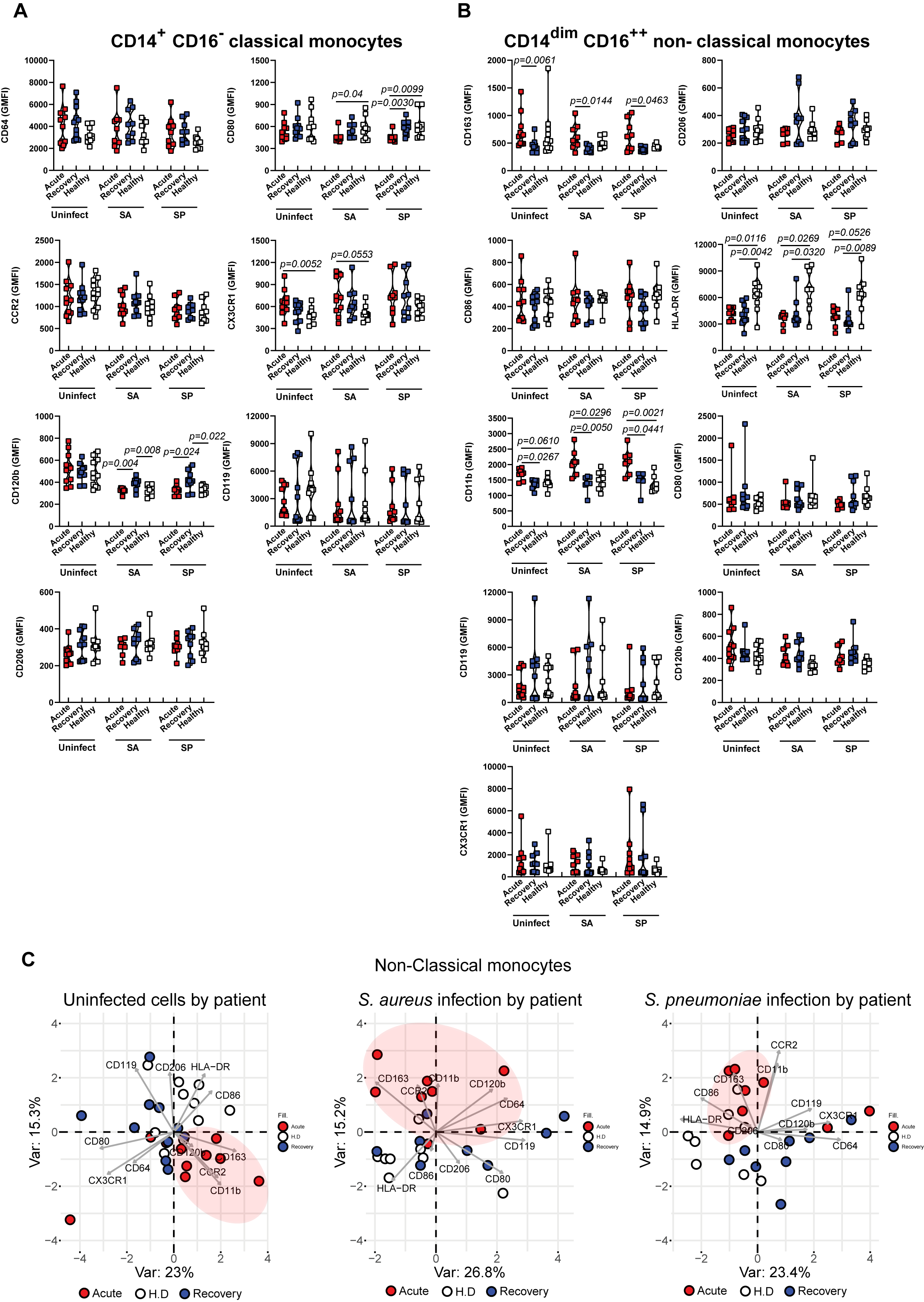

Supplement: S8 Fig — (A and B) Flow cytometric determination of the expression of key surface markers in CD14+ CD16- classical (A) and CD14dim CD16+ non-classical monocytes (B) from COVID-19 acute- (red) or rec-phase (blue) as well as healthy donors (white), pre-exposed to 10% COVID-19 plasma (solid symbols) or healthy donor plasma (open symbols) for 3 h and subsequently challenged with SA or SP at MOI 1 or left unchallenged. (C) PCA of cell surface phenotype of COVID-19 patients acute- (red) or rec-phase (blue) and healthy donors’ (white) non-classical monocytes at the basal level without bacterial challenge, upon SA infection or SP infection. Each symbol represents cells from a single COVID-19 patient or healthy donor exposed to either COVID-19 or healthy donor plasma from one donor. For panels A and B, p values were determined by using Mann-Whitney test. (TIF) [file ppat.1010176.s014.tif]

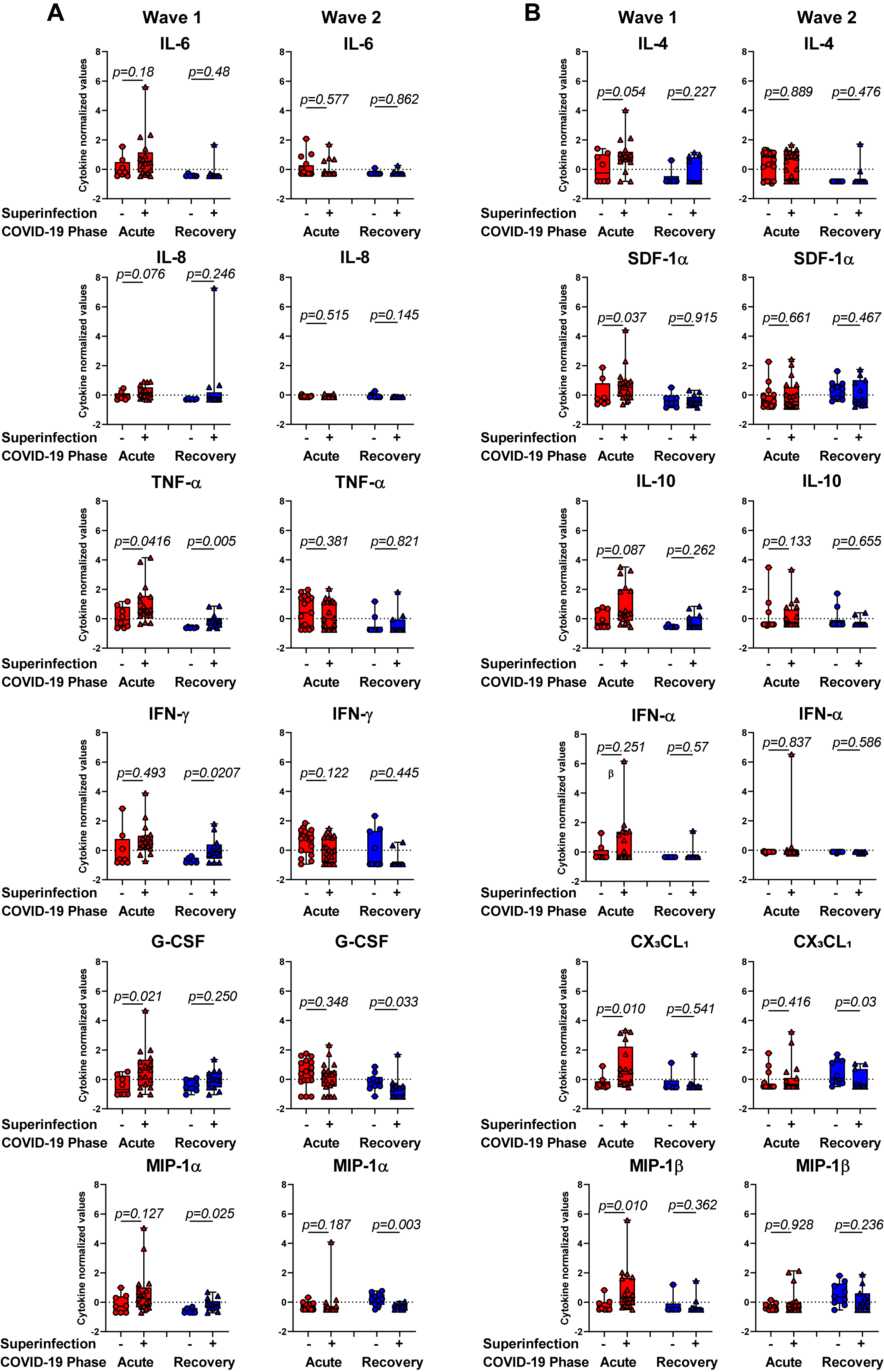

Supplement: S9 Fig — (A and B) Normalized cytokine values (sum of Z-scores) in the plasma of acute- (red) and rec-phase (blue) patients with (triangle) or without (circle) bacterial superinfection from first wave left) and second wave (right). Data presented as whisker plots with box indicating interquartile range and error bars indicating highest and lower value. For panels A-B, p values were determined by using Mann-Whitney test. (TIF) [file ppat.1010176.s015.tif]

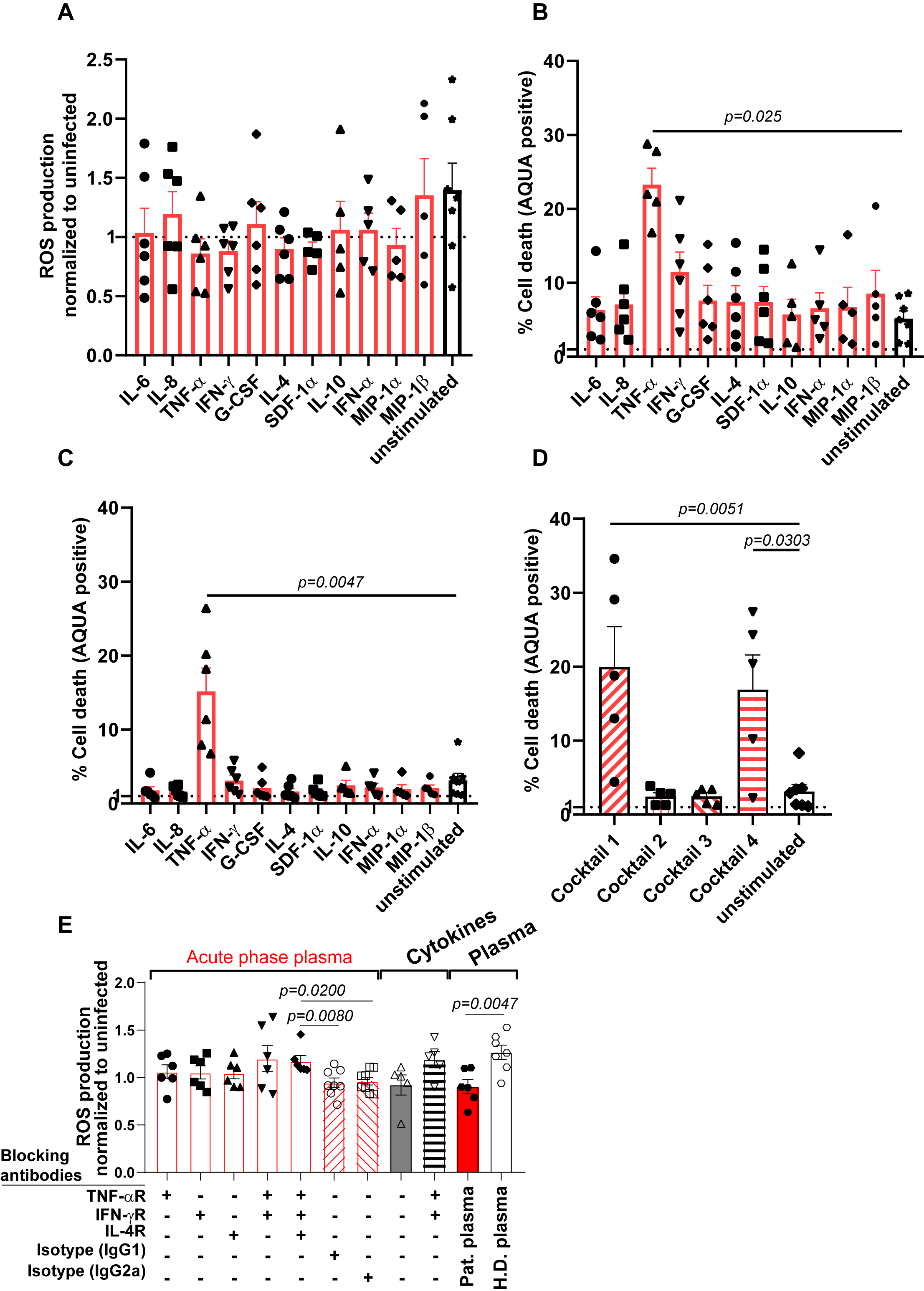

Supplement: S10 Fig — ROS production (A) and cell death (B) of healthy donor neutrophils (n = 5–7) pre-exposed to the indicated single (A, B, C) or combination of cytokines for 4 h (D) and subsequently infected with SA at MOI 1 (A and B) for 2 h. (C and D) Cell death of healthy donor neutrophils (n = 5–7) only exposed to the indicated single (A) or combination of cytokines for 4 h (D). Cocktail 1, TNF-α and IFN-γ; cocktail 2, IL-6, IL-8, G-CSF and IFN-α; cocktail 3, IL-4, IL-10 and IFN-γ; cocktail 4, IL-4, IL-6, IL-8, IL-10, TNF-α, IFN-α, IFN-γ, G-CSF, SDF-1α, MIP-1α and MIP-1β. Each symbol represents neutrophils from one healthy donor. Data are presented as the mean value ± SEM. (E) ROS production of healthy donor neutrophils (n = 5–8) pre-treated with receptor blockers or isotype controls and exposed to 10% acute-phase COVID-19 plasma (“Acute-phase plasma”, n = 6 plasmas). Employment of receptor blockers or isotype controls, pre-exposed to TNF-α and IFN-γ (“Cytokines”) with or without receptor blockers and 10% acute-phase COVID-19 or 10% healthy donor plasma (“Plasma”, n = 6 plasmas) for 4 h and subsequently infected with SA at MOI 1. Each symbol represents cells from one healthy donor. For graphs presented in B-D, p values were determined by using Mann-Whitney test, for E, by using Kruskal-Wallis test with Dunn’s multiple comparison test and Mann-Whitney test. (TIF) [file ppat.1010176.s016.tif]

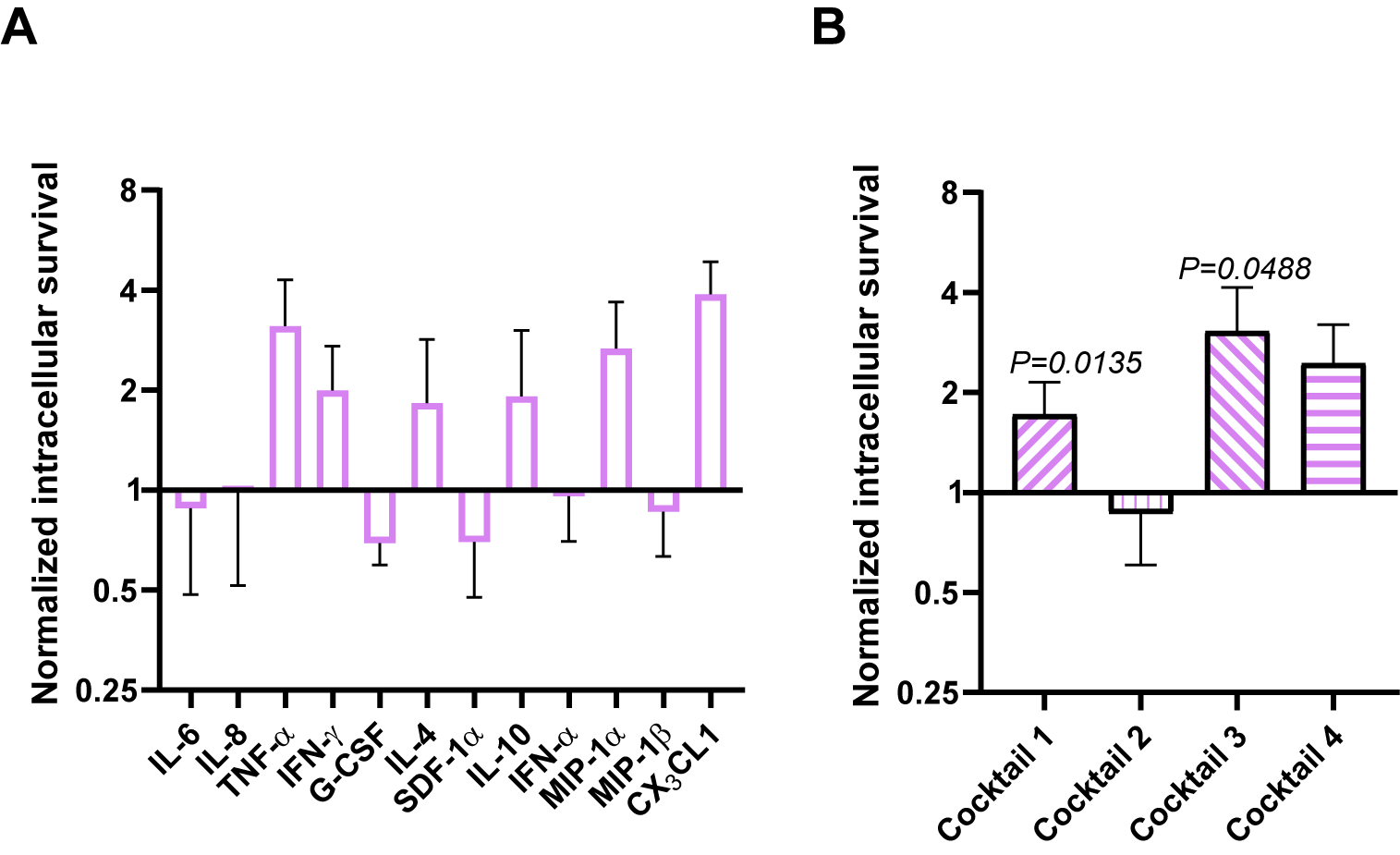

Supplement: S11 Fig — Intracellular bacterial survival in healthy donor monocytes (n = 9) pre-exposed to the indicate single (A) or combination of cytokines for 18 h (B) and subsequently infected with SA at MOI 10 (A and B) or 1 (C). Cocktail 1, TNF-α and IFN-γ; cocktail 2, IL-6, IL-8, G-CSF and IFN-α; cocktail 3, IL-4, IL-10, IFN-γ and CX3CL1; cocktail 4, IL-4, IL-6, IL-8, IL-10, TNF-α, IFN-α, IFN-γ, G-CSF, SDF-1α, MIP-1α, MIP-1β and CX3CL1. For panels A and B p values were determined by using Mann-Whitney test. (TIF) [file ppat.1010176.s017.tif]

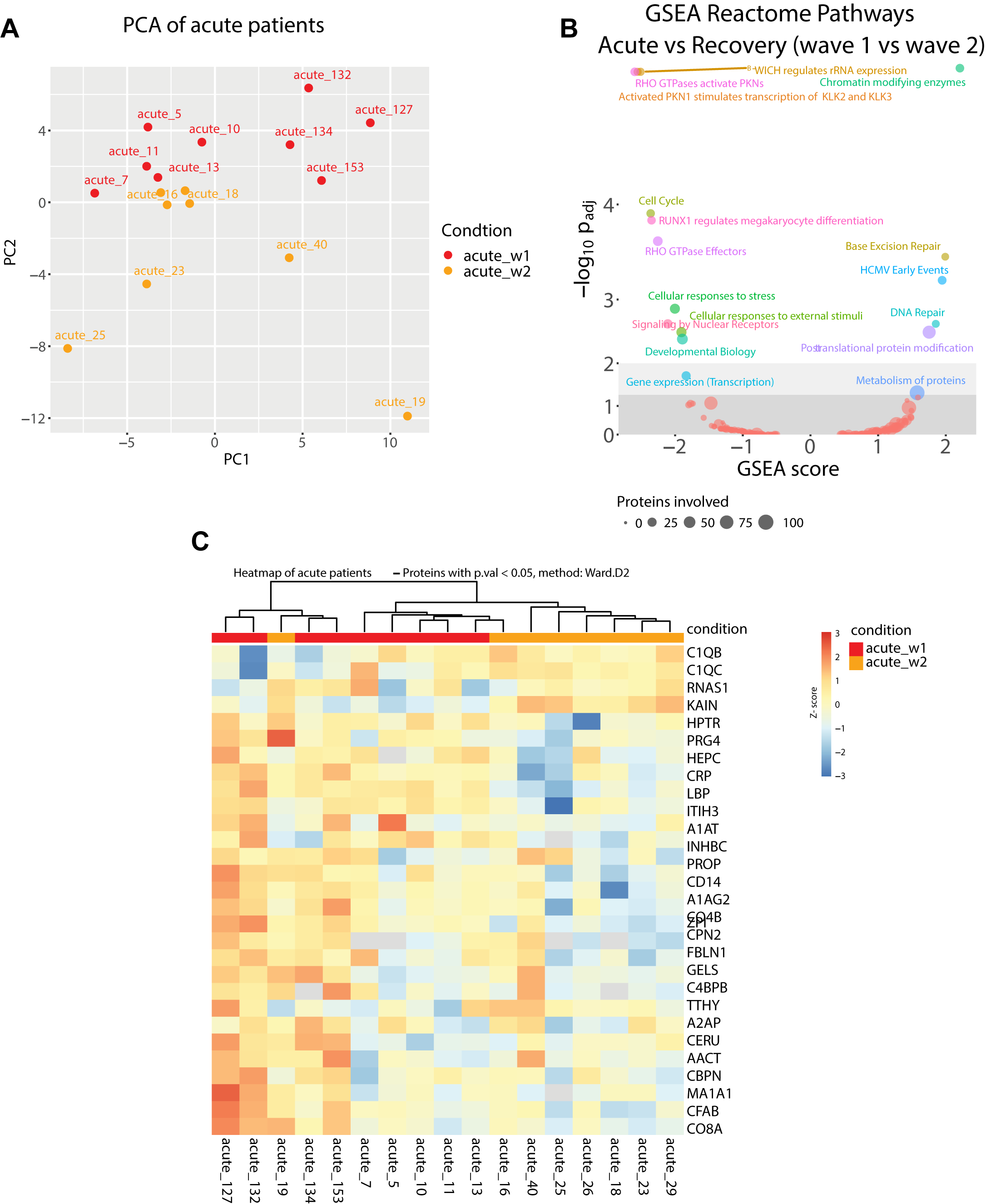

Supplement: S12 Fig — (A) PCA analysis of the proteomic profiles from nine acute patients from wave 1 and eight acute patients from wave 2 and (B) changes in the representation of plasma proteins and their impact on GSEA analysis of metabolic and cellular immune defense pathways between acute- and rec-phase wave 1 vs acute- and rec-phase wave 2. A p-value of 0.05 and a minimum change of two folds and an FDR of 0.05 and 0.25 was considered for statistical significance. (C) heat map of proteins presenting significant changes in their intensity and classified by the patient status (acute- or rec-phase) and the time of sampling (wave 1 or wave 2). A p-value of 0.05 was considered for statistical significance and no FDR was used for the heat map determination. (TIF) [file ppat.1010176.s018.tif]

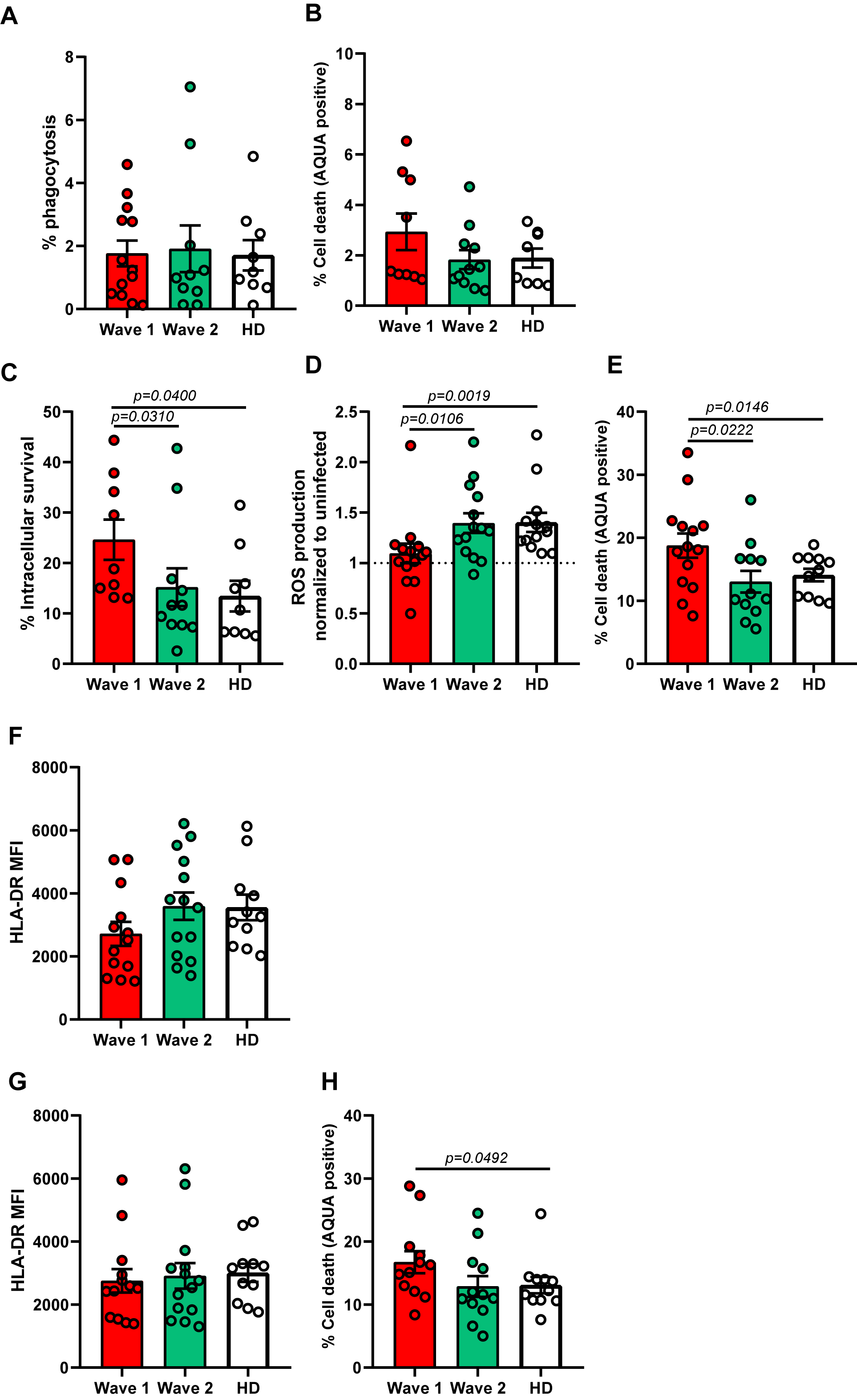

Supplement: S13 Fig — (A) Phagocytosis capacity of healthy donor neutrophils (n = 6) pre-exposed to 10% of wave 1 (n = 12), wave 2 (n = 11) or healthy donor plasma (n = 9) for 4 h and subsequently challenged with SA at MOI 10. (B) Cell death of healthy donor neutrophils (n = 5) pre-exposed to 10% of wave 1 (n = 9), wave 2 (n = 11) or healthy donor plasma (n = 8) for 4 h. (C-F) Intracellular bacterial survival (C), ROS production (D), cell death (E) and HLA-DR expression (F) of healthy donor monocytes (n = 7) pre-stimulated with 10% of wave 1 (n = 12–14), wave 2 (n = 12–14) or healthy donor plasma (n = 11–12) and subsequently infected with SA. (G and H) HLA-DR expression (G) and cell death (H) of healthy donor monocytes (n = 7) pre-stimulated with 10% of wave 1 (n = 12), wave 2 (n = 12) or healthy donor plasma (n = 11) uninfected cells. (TIF) [file ppat.1010176.s019.tif]

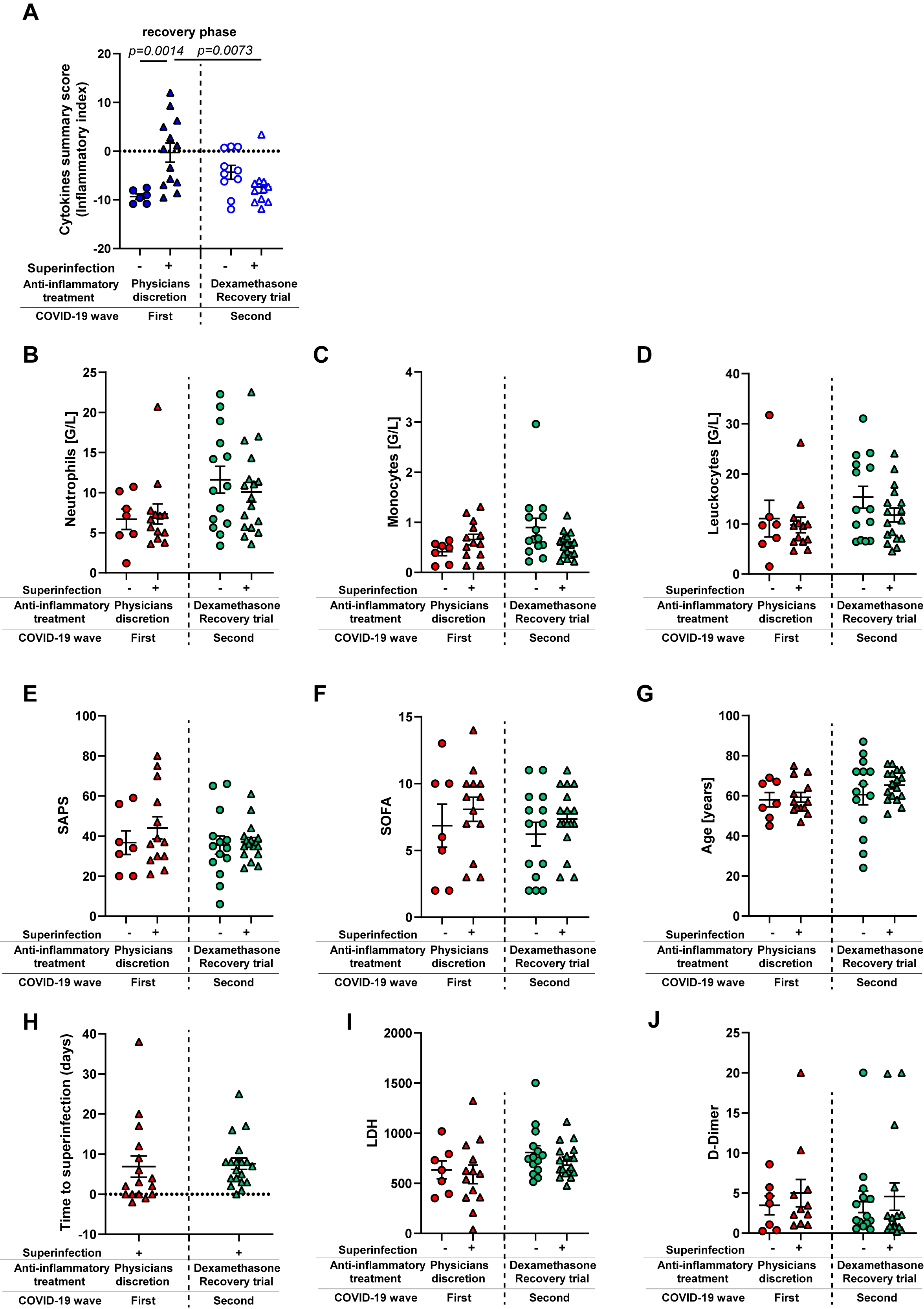

Supplement: S14 Fig — (A) Normalized cytokine values (sum of Z-scores) depicted as cytokine summary score (inflammatory index) in the plasma of rec-phase COVID-19 patients between first and second wave with or without superinfection. The cytokine summary score from first wave is also presented in Fig 1D. (B-I) Clinical as well as laboratory parameters that are part of the routine diagnostics including neutrophils (B), monocytes (C) and leukocytes counts (D), SAPS (E) and SOFA score (F), age (G), time to superinfection (H), LDH (I) and D-dimer levels (J) in acute-phase COVID-19 patients during first (n = 25) vs second wave (n = 38), with or without superinfection. Data are presented as the mean value ± SEM. SAPS, Simplified acute physiology score; SOFA, Sepsis-related organ failure assessment score; LDH, lactate dehydrogenase. P values determined using Mann-Whitney test. (TIF) [file ppat.1010176.s020.tif]

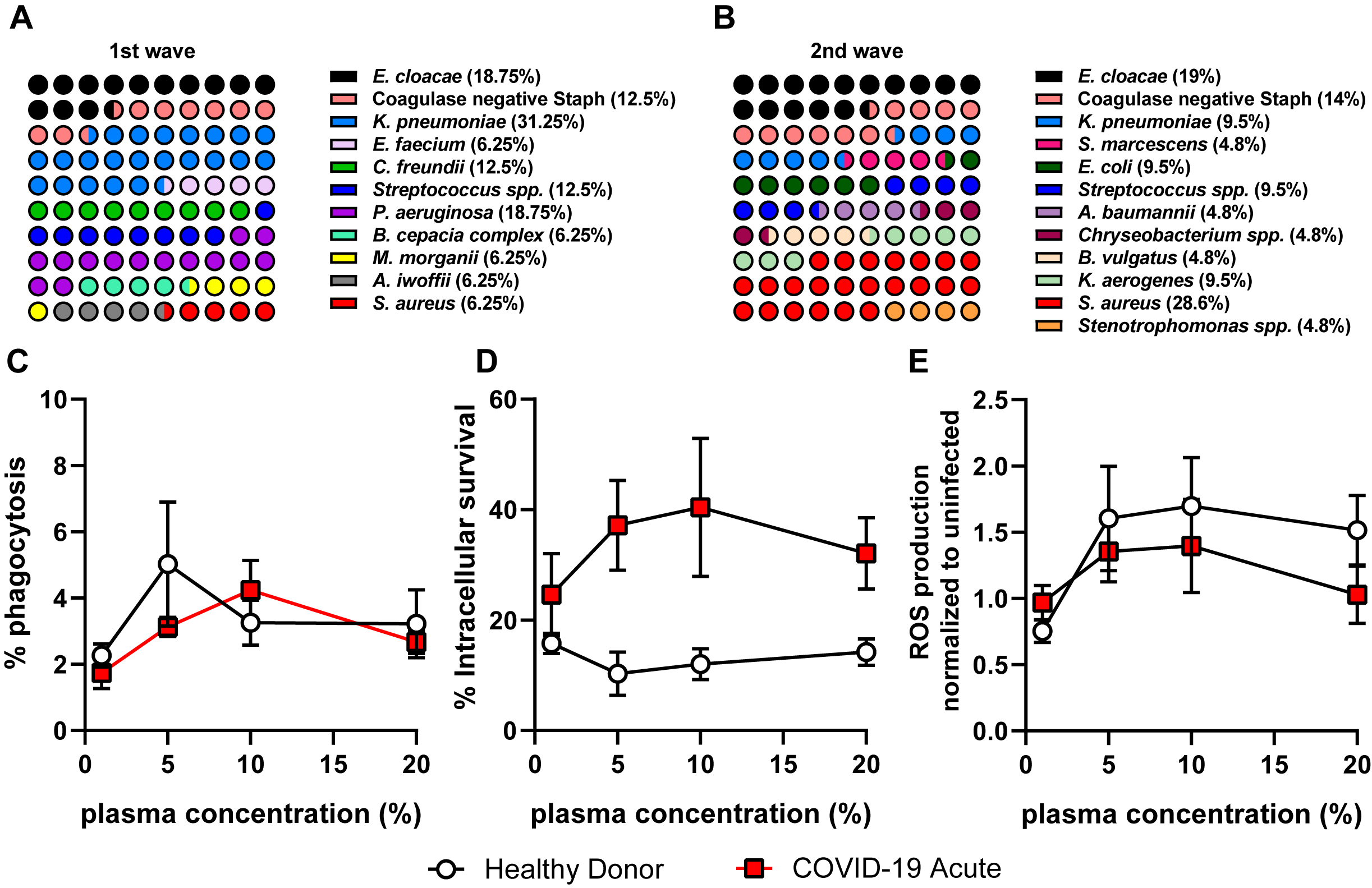

Supplement: S15 Fig — (A and B). Percentage of identified species in bacterial superinfection cases among critically ill COVID-19 patients during wave 1 (A) and wave 2 (B). (C-E) Plasma titration assay using COVID-19 acute-phase or healthy plasma at 1, 5, 10 or 20% in RPMI and neutrophils from healthy donors. Phagocytosis (A), intracellular survival (B) and ROS production (C) after infection with Staphylococcus aureus were determined from the different stimulations with plasma. (TIF) [file ppat.1010176.s021.tif]
